# Prediction of the classification, labelling and packaging regulation H-statements with confidence using conformal prediction with N-grams and molecular fingerprints

**DOI:** 10.1016/j.crtox.2025.100242

**Published:** 2025-05-22

**Authors:** Ulf Norinder, Ziye Zheng, Ian Cotgreave

**Affiliations:** a Department of Computer and Systems Sciences, Stockholm University, P.O. Box 1073, SE-164 25 Kista, Sweden; b MTM Research Centre, School of Science and Technology, Örebro University, 701 82 Örebro, Sweden; c Cytiva, Björkgatan 30, 75 323 Uppsala, Sweden; d Chemical and Pharmaceutical Safety, Research Institute of Sweden (RISE), Forskargatan 18, 15 136 Södertälje, Sweden; e IVL Swedish Environmental Research Institute, 10 031 Stockholm, Sweden

**Keywords:** CLP Regulation, Conformal prediction, Consensus modeling, H-statements, Molecular fingerprints, N-grams, Random forest

## Abstract

Effective chemical hazard labelling systems are essential for safeguarding human health and the environment as a result of widespread chemical use, and machine-learning models can be used to predict hazard labels efficiently and reduce the use of animal tests. This investigation shows the utility of N-grams and other fingerprint featurization procedures for predicting classification, labelling and packaging (CLP). Regulation H-statements, particularly in an ensemble (consensus) setting. Consensus modelling by class or Conformal Prediction median p-values seems to be particularly advantageous in order to obtain both high conformal prediction validity and efficiency as well as good balanced accuracy, sensitivity and specificity. Utilization of the N-grams allows handling of all symbols in SMILES strings including those related to metals and salts that may be important for the compounds to exhibit their experimental determined toxicities. The models developed in this study are efficient tools to access hazard classification H-statements of chemicals, which can be useful for chemical hazard assessment, read-across as well as risk management.

## Highlights

• Predictive models were developed for H-statement predictions.
• Consensus models were found to be the best models.
• Stringent and robust uncertainty and error handling for each class using Conformal Prediction.
• Stringently defined applicability (reliability) domains for the developed models.
• N-gram characterization of SMILES enables treatment of all characters, including metals and ions, found in SMILES.

## Graphical abstract

**Figure:**
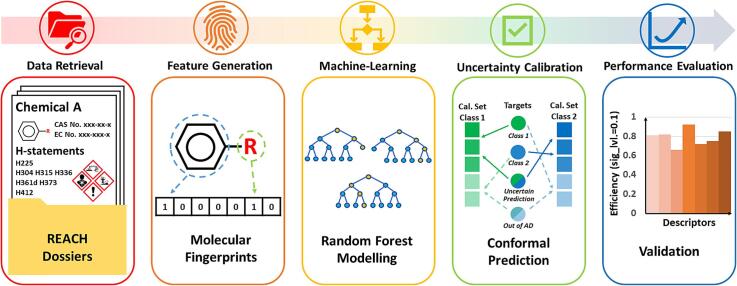

## Introduction

Chemical substances and mixtures can pose various hazards, including physical hazards such as flammability or reactivity, health hazards like toxicity or carcinogenicity, and environmental hazards like aquatic toxicity or unwanted behaviors such as bioaccumulation, respectively. To mitigate risks arising from exposure, effective and standardized hazard communication systems are critical. These systems help protect workers, consumers, and the environment by providing clear and consistent information about the potential hazards of chemicals, and reduce the risk of accidents, exposures, and environmental damage. In response to the global need for consistent chemical safety standards to address the issue of chemical exposure and to protect human and environmental health, the United Nations (UN) founded an internationally recognized, harmonized system of chemical classification and labelling known as Globally Harmonized System (GHS) (UNECE, 2003, UNECE, 2024). Later, the European Union (EU) adopted GHS framework in the form of Classification, Labelling and Packaging (CLP) regulation (EC No 1272/2008), which became mandatory in 2009 (Commission Regulation, 2008). The CLP regulation plays a crucial role in ensuring the safety of chemicals within the EU market. It requires manufacturers, importers and distributors to classify the hazards of substances and mixtures before placing them on the market, ensuring that labels, packaging, and safety data sheets reflect the correct hazard information. This regulation serves as the cornerstone of chemical safety across Europe, promoting informed decision-making, regulatory compliance, and risk management across a range of industries.

One of the key elements of the CLP regulation is the use of H-statements (hazard statements), which replaced the R-phrases under the GHS adoption. These statements communicate specific risks a chemical can cause during its handling, use, transport or storage. The H-statements are grouped into three categories: Physical hazards, Health hazards and Environmental hazards (Commission Regulation H-statements). The physical hazards are defined based on chemical reactivity such as explosivity, flammability, self-heating, oxidization, and corrosivity, whereas health and environmental hazards are classified based on laboratory testing, covering endpoints such as acute toxicity, carcinogenicity, skin sensitization and acute aquatic toxicity etc. In general, H-statements are denoted in the form of a alphanumeric code that begins with a letter “H” followed by three digits code numbering (e.g. “H300” indicates oral acute toxicity) (De Groot et al., 2010). These codes are essential in hazard communication as they appear on chemical labels and safety data sheets, ensuring that all stakeholders—including workers, emergency responders, consumers, and regulatory bodies—have access to standardized and reliable information regarding chemical hazards. The implementation of CLP in the EU is significant because it aligns chemical safety with global standards while ensuring compliance with local laws, creating a safer work environment and reducing chemical-related incidents. The H-statements (ECHA CLP) have also been widely used in chemical risk management studies, from early-phase hazard screening to a robust life cycle assessment of consumer products. For example, ProScale, a hazard and exposure-based scoring system widely used in life cycle assessments, uses CLP H-statements to characterize chemical toxicity for both human toxicity (Rydberg et al., 2021) and ecotoxicity (ProScaleE; Halling, 2024). The hazard communication system of CLP enables informed decision-making, risk management and regulatory compliance across diverse industries and countries, ultimately supporting safer chemical handling, transport, and use, contributing to overall public health and environmental protection.

The CLP regulation requires manufacturers, importers and distributors to compile and evaluate all available data related to the hazardous properties of a substance to ensure accurate classifications in the labels and safety data sheets. Where data is either inadequate or not of sufficient quality to serve the classification purpose, additional laboratory tests may be performed in compliance with the REACH Regulation and OECD guidelinces. Notably, The CLP regulation upholds the ethical standards outlined in the EU DIRECTIVE 2010/63/EU (European Parliament, 2010), stating that testing on humans and non-human primates is strictly prohibited, and new testing is only conducted when absolutely necessary (European Parliament, 2010). In other words, despite the robustness of the CLP framework, a significant challenge arises when dealing with novel chemicals with insufficient safety data, as a lack of proper H-statements classifications can lead to miscommunication and increase risks of possible chemical exposures. Therefore, there is an increasing demand for novel tools that can efficiently assess the hazard classifications of chemicals that do not rely on animal testing.

In this study, we have developed a non-animal and machine-learning based computational approach for predicting H-statements classification of substances. The H-statement data used in this study were retrieved from the European Chemicals Agency (ECHA) REACH registration dossier. The REACH registration dossier is a publically available database with entries of chemical information according to the REACH regulation. Compared to other regulatory data sources such as the CLP annex VI and the safety datasheets which only contains the active H-statements, the REACH registration dossier also provides information when compounds can be conclude as inactive (non-toxic) for certain H-statement endpoints, thus more suitable for model training. While machine learning methods have been widely applied to predict some toxicity endpoints, such as mutagenicity, many previous studies have focused on similar or overlapping literature databases. This can limit the diversity of the data and reduce the generalizability of the models. In contrast, our study utilizes the EU regulatory-compliant data source (the REACH registration dossier) that has only been explored for building machine learning models for a few endpoints, for example reproductive toxicity (Liu et al., 2023) and eye irritation (Luechtefeld et al., 2016). By leveraging the REACH registration dossier, we aim to expand the scope of predictive models to a much broader range of H-statement endpoints for human health and environmental hazards. The regulatory relevance of the data from REACH dossier is a significant advantage, as it complies with official guidelines and is trusted by both industries and authorities, making it more applicable for real-world regulatory assessments in EU. With the retreived data, our objective was to develop appropriate in silico models using a random forest algorithm for predicting H-statements. In addition, we have incorporated to the use of Mondrian conformal prediction into the approach, in order to provide statistical appraisal of uncertainty in the model predictions.

## Methods

### Data retrieval

In the REACH registration dossier, each entry has a unique numerical registration number. The largest registration number, at the time of search (March 23rd, 2023), was 34,597 and in this study, we searched for information with potential registration numbers ranging from 0 to 40,000 to make sure all entries in the REACH registration dossiers were extracted. Python Beautiful Soup code was used to identify html elements on the respective webpages. The chemical identities (EC Number, EC Name, CAS Number, Molecular formula and IUPAC Name) and “Type of substance” were extracted from the “General Information**-**Substance identity” page (“https://echa.europa.eu/sv/registration-dossier/-/registered-dossier/x/1/1”, where x is the registration number), and substance registration details were retrieved from the “General Information-Administrative information” page (“https://echa.europa.eu/sv/registration-dossier/-/registered-dossier/x/1/2”). The H-statement chemical hazard labels according to the CLP regulation were downloaded from the “Classification & Labelling & PBT Assessment-GHS” page (“https://echa.europa.eu/sv/registration-dossier/-/registered-dossier/x/2/1”) under the “classification” html element. The H-statement labels include physical hazards (H2xx), health hazards (H3xx) and environmental hazards (H4xx). Health hazards and environmental hazards were included in this study.

For pseudo code, see Supplementary Material.

### Datasets

#### Investigated H-statements

The following procedure for data selection was performed for each of the investigated groups of H-statements (for a list, see Table 1).

**Table 1:** H-statement groups and included H-statements and number compounds in each class.

**H-statement group**	**Included H-statements**	**Corresponding endpoints**	**#cmpds H-statement class**	**#cmpds non-H-statement class**
H30x012345	H300, H301, H302, H303, H304. H305	Fatal or harmful if swallowed	4481	3108
H31x0123	H310, H311, H312, H313	Fatal or harmful in contact with skin	1067	5942
H31x57	H315, H317	Skin irritation	4931	5342
H33x0123	H330, H331, H332, H333	Fatal or harmful if inhaled	393	8433
H33x45	H334, H335	Respiratory irritation	1708	1994
H34x	All subcategories of H34	Genetic toxicity	393	8433
H35x	All subcategories of H35	Carcinogenicity	510	2769
H36x01	H360x,H361x	May damage fertility or unborn child	903	4905
H40x	All subcategories of H40	Toxic to aquatic life	1754	7615
H41x	All subcategories of H41	Toxic to aquatic life with long lasting effects	4847	5040

For the retrieved data records, only substances with chemical information that can be linked to a single compound with clear structure information (SMILES) are used. Other records such as mixtures, polymers, or confidential substances with no clear structural information are discarded. The compound was labelled as H-statement active if the H-statement was found at least one time. For the remaining compounds, a compound was labelled as H-statement inactive if the statement “data conclusive but not sufficient for classification” was found at least one time. Other compounds that were registered in the REACH dossiers but cannot be labelled as H-statement active or inactive due to lacking or conflicting data were discarded.

Table 1 shows the groups of H-statements for which models have been developed.

#### Dataset standardization and splitting

The datasets SMILES (Weininger, 1988) were standardized using RdKit (2023.9.4) (RDKit) function MolFromSmiles(smi,sanitize = True).

Scikit-learn (1.1.3) cross-validation (KFold) was used to create 10 folds (10-fold CV) (Pedregosa et al., 2011). A 20 %, randomly selected calibration set was also generated from the respective training set of each fold for the Conformal Prediction analysis (see Section”Conformal Prediction” for more details).

### Descriptor generation and machine learning methods

In this study, we use several different molecular fingerprints as well as the N-gram method to describe chemical features. Molecular fingerprints are widely used in cheminformatics to represent chemical structures in a compact and computationally efficient manner. They are binary or numerical vectors that encode the presence or absence of specific molecular features, such as substructures, functional groups, or atom connectivity patterns. These fingerprints simplify complex molecular structures into a form that can be easily processed by machine learning algorithms, facilitating tasks like similarity searches, clustering, and predictive modelling. N-gram methods are commonly used in natural language processing but have also been adapted for cheminformatics to represent chemical structures. In this approach, chemical structures are broken down into continuous sub-sequences of atoms or bonds, typically called “fragments” or “chemical fingerprints.” These N-grams, where “N” represents the length of the fragment, capture local structural patterns within molecules. By encoding the presence or absence of these substructures, N-grams provide a simplified yet informative representation of chemical compounds.

#### N-grams

In this study, the N-grams was generated in the following manner:

1. A dictionary was used

{'a': 101, 'b': 102, 'c': 103, 'd': 104, 'e': 105, 'f': 106, 'g': 107, 'h': 108, 'i': 109, 'j': 110, 'k': 111, 'l': 112, 'm': 113, 'n': 114, 'o': 115, 'p': 116, 'q': 117, 'r': 118, 's': 119, 't': 120, 'u': 121, 'v': 122, 'w': 123, 'x': 124, 'y': 125, 'z': 126,'A': 127, 'B': 128, 'C': 129, 'D': 130, 'E': 131, 'F': 132, 'G': 133, 'H': 134, 'I': 135, 'J': 136, 'K': 137, 'L': 138, 'M': 139, 'N': 140, 'O': 141, 'P': 142, 'Q': 143, 'R': 144, 'S': 145, 'T': 146, 'U': 147, 'V': 148, 'W': 149, 'X': 150, 'Y': 151, 'Z': 152, ' ': 153, '\\': 154, '/': 155, '.': 156, '[': 157, ']': 158, '(': 159, ')': 160, '=': 161, '#': 162, '@': 163, '0′: 164, '1′: 165, '2′: 166, '3′: 167, '4′:68, '5′: 169, '6′: 170, '7′: 171, '8′: 172,'9′: 173, '+': 174, '-': 175, '%': 176, '|': 177}.

2. An N-gram length of 4 (or 6) was used and slided one position to the right from start to end of the investigated SMILES string. The 4 characters of each 4-gram were assigned the corresponding dictionary number and hashed onto the given hash length. For example a string, e.g. smiles, such as abcdag the first 4-gram abcd is assigned a number 101102103104. The next string, moved one position to the right, bcda is encoded as 102103104101 and the last 4-gram cdag as 103104101107. Each of these 3 encoded numbers are then hashed onto the given hash length, e.g. 1024, by the modulo operator to produce positions 576, 613 and 755, respectively. Counts of hash length positions are used, e.g. if position 576 is already assign a “1” then a new n-gram producing a hashed 576 position would increment this number by 1 for a result of 2 and so on. The corresponding process was performed for the 6-grams.

Hashing lengths of 64, 256 and 1024 were used.

### Core-substituent fingerprints

Core-Substituent Fingerprints (Core-Substituent_fps) were calculated using parts of TISBE (https://github.com/f48r1/TISBE, accessed Jan 15, 2024) (Mastrolorito et al., 2024) A binary fingerprint (1/0) of length 1000 was generated.

### SMILES Extended connectivity fingerprints

SMILES Extended Connectivity Fingerprints (SMILES_extnd_connect_fps) were calculated using parts of MHFP (https://github.com/reymond-group/mhfp, accessed March 9, 2024)) (Probst and Reymond, 2018). A binary fingerprint (1/0) of hashed length 1024 was generated. SMILES Extended Connectivity Fingerprints radii of 2 and 4 were used, respectively.

### Morgan fingerprints

Morgan fingerprints were calculated using the RdKit (2023.9.4) (RDKIT) function GetMorganFingerprint (mol, radii, useChirality = True, useCounts = True), using a fingerprint hashed length of 1024 and radii of 2 and 4, respectively.

### N-GramsPE

The N-GramsPE were generated using SmilesPE (0.0.3, https://github.com/XinhaoLi74/SmilesPE, accessed June 10, 2021) (Li and Fourches, 2021) patterns followed by K-merization using a N-gram length of 4 and a hashing length of 1024.

### DeepSmiles N-grams

DeepSmiles (O’Boyle and Dalke, 2018) N-Grams were generated using DeepSmiles (version 1.0.1) with the deepsmiles.Converter(rings = True, branches = True), followed by K-merization, using a N-gram length of 4 and a hashing length of 1024.

### SELFIES N-grams

SELFIES (Krenn et al., 2020) N-Grams were generated using SELFIES (version 1.0.4) with the selfies.encode function followed by K-merization using a N-gram length of 4 and a hashing length of 1024.

### Machine learning

The N-gram and molecular fingerprint representations were analysed using the Scikit**-**learn **(**1.1.3**) (**Pedregosa et. al., 2011) RandomForestClassifier, with default settings, as base classifier embedded within the Conformal Prediction framework.

### Conformal prediction

Conformal prediction (CP) is a mathematical framework, based on a mathematical proof, developed by Vovk and co-workers (Vovk et al., 2005, Balasubramanian et al., 2014) that guarantees an error rate set by the user, provided that the data is *exchangeable*. CP Is a post-processing step after the model building and performs a re-calibration of the test data prediction output using an independent calibration set, usually randomly selected from the training set before the model building. Conformal prediction was chosen for this study because it provides a flexible and rigorous framework for quantifying the uncertainty of machine learning model predictions. Unlike traditional methods, which may provide a point estimate with no accompanying measure of reliability, conformal prediction offers a statistically valid way to associate each prediction with a confidence level (Shafer and Vovk, 2008). This is particularly important in chemical hazard prediction, where misclassifications can lead to serious health and environmental risks. By using conformal prediction, we can produce prediction intervals or confidence regions that indicate how reliable each prediction is, offering greater transparency and interpretability for regulatory decisions. Compared to other uncertainty quantification methods, such as Bayesian approaches, conformal prediction does not require complex assumptions about the underlying data distribution (Papadopoulos, 2008) making it more computationally efficient and easier to implement across diverse datasets. Moreover, the use of Mondrian conformal prediction allows for class-conditional confidence estimates, which are crucial in multi-label classification tasks such as predicting multiple H-statement categories. This method enhances model performance by providing not just accurate predictions, but also calibrated measures of uncertainty, thereby ensuring more reliable hazard classifications.

The Mondrian version of CP that performs the re-calibration separately on each class in the data set, which guarantees the error rate in a class-wise manner, was employed in this work.

Scikit-learn (version 1.1.3) was used for building the underlying random forest models (RandomForestClassifier) and nonconformist (version 1.2.5, https://github.com/donlnz/nonconformist, accessed January 28, 2021) for building the Mondrian conformal predictors. Default parameters were used.

The CP binary classification produces one of 4 possibilities as output: A single label prediction for either of the 2 classes, both labels (*both* class*)* or no label (*empty* class). *Validity* and *Efficiency* are 2 important outcome parameters for quantifying the quality of the derived CP models. *Validity* (for each class for Mondrian conformal predictors) is the percentage of correct predictions at the user set significance level (percentage of acceptable errors). Here it should be observed that the *both* prediction is always a correct prediction for binary classification as it always contains the correct class while the *empty* prediction is always erroneous since it contains no classes.

*Efficiency* (for each class for Mondrian conformal predictors) is the percentage of single label class predictions, regardless of whether these predictions are correct or not.

For a more detailed description of the CP procedure, see reference (Norinder et al., 2018).

The aim is therefore to obtain as high, informative, percentage single label class predictions as possible, i.e. high efficiency, at the significance level set by the user.

### Consensus modelling

Two alternatives for consensus modelling were performed; one based on the resulting median CP p-value for each compound and for each class from all models (consensus_pvals_xx) at a particular significance level and the other alternative based on defining the predicted class for each compound based on a majority vote by all included models (consensus_cls_xx) at the investigated significance level. For equal numbers of maximum votes at the investigated significance level the order of assigning the class was the following: H-statement labelled class > non-H-statement labelled class > both class > empty class.

Three different descriptor sets were used:

All sets
Selected set1: Core-Substituent_fps, deepsmiles, morgan2, ngramsPE, SMILES_extnd_connect_fps_2, selfies and ngram_4_hashed_1024
Selected set2: ngram_4_hashed_64, ngram_4_hashed_256, ngram_4_hashed_1024, ngram_6_hashed_64, ngram_6_hashed_256, ngram_6_hashed_1024 and ngramsPE

### Performance metrics

Apart from the CP performance metrics *Validity* and *Efficiency* mentioned in section”Conformal Prediction” additional, more traditional, metrics such as accuracy (Acc), balanced accuracy (BA), sensitivity (SE), specificity (SP) and Matthews's correlation coefficient (MCC) were also calculated from the CP single label predictions as well as ROC_auc from p-values differences (p_value_H-statement_class – p_value_non-H-statment_class) (see Table “H-statements_statistics” in Supplementary Material).

## Results and discussion

### Computational efforts

All fingerprints and N-grams are fast to generate and completed within a few minutes or less for the datasets studied in Table 1.

### Model performance

#### Overall model performance

**Fig. 1:**
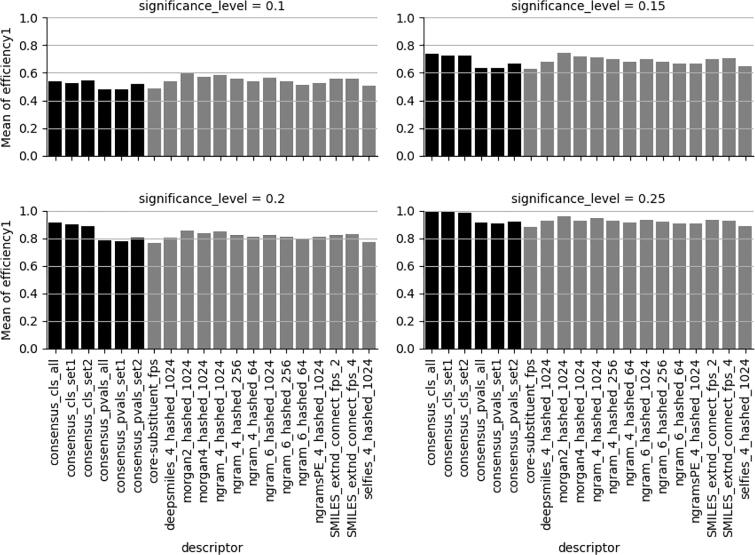
Efficiencies for the H-statement labelled class at different significance levels (0.1, 0.15, 0.2, 0.25). Consensus models in black. The descriptor nomenclature is as follows: fingerprint-type followed by the length or radius for the fingerprint followed by the hashed fingerprint length, e.g. deepsmiles_4_hashed_1024, and for the consensus models: consensus followed by based on predicted class (cls) or CP p-values (pvals) and the set of models used (see section “Consensus Modelling” for definitions of set1 and set2).

**Fig. 2:**
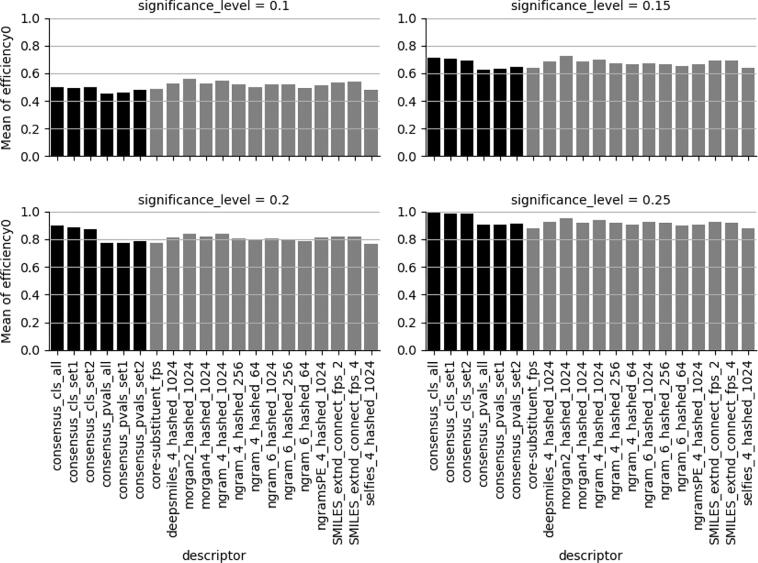
Efficiencies for the non-H-statement labelled class at different significance levels (0.1, 0.15, 0.2, 0.25). Consensus models in black. See Fig. 1 for an explanation of descriptor and concensus models nomenclature.

The results presented in Fig. 1, Fig. 2 show that most methods perform reasonably well on the investigated datasets with respect to efficiency. At a significance level of 0.2, i.e. accepting 20 % errors, the majority of the models show efficiencies close to or above 80 % indicating that the single label predictions, containing only one class label, are close to 80 % or better. This, in turn, provides a reasonably good basis for making decisions regarding whether a set of investigated compounds will be given a single H-statement label or not.

**Fig. 3:**
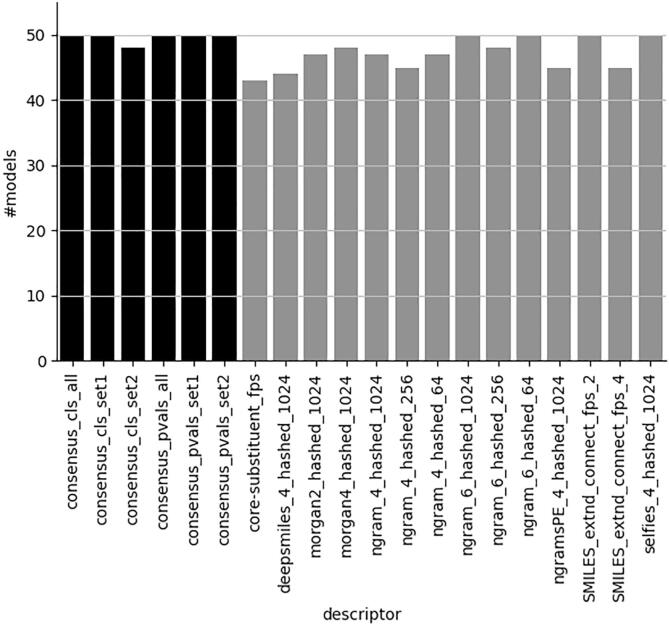
Number of valid models across all significance levels. (0.1, 0.15, 0.2, 0.25, 0.3). Maximum number is 50. Consensus models in black. See Fig. 1 for an explanation of descriptor and concensus models nomenclature.

Fig. 3 shows that the different approaches, i.e. different descriptors and consensus modelling, are able to develop *valid* models for all, or a vast majority, of the investigated CLP categories across the significance levels, e.g. 0.1, 0.15, 0.2, 0.25 and 0.3.

**Fig. 4:**
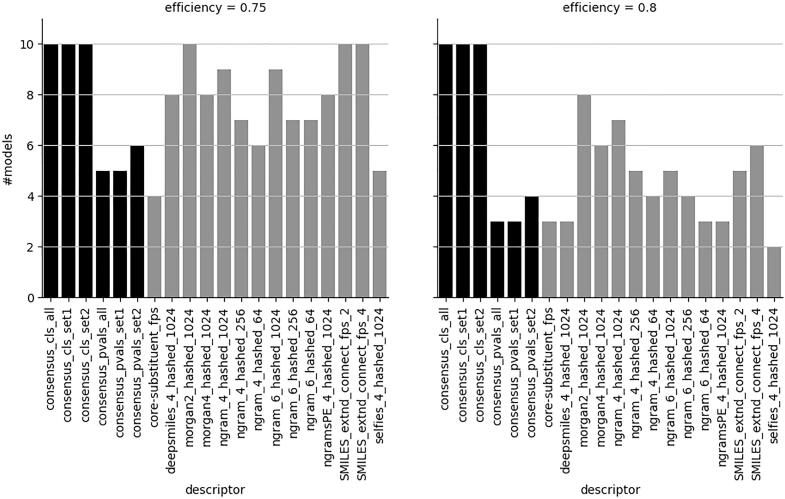
Number of valid models at significance level 0.2 with efficiencies of both classes ≥ 0.75 and 0.8, respectively. Maximum number is 10. Consensus models in black. See Fig. 1 for an explanation of descriptor and concensus models nomenclature.

However, when forcing the models to have at least 80 % efficiency for both classes these criteria are first met at the 0.2 significance level, and Fig. 4 shows that the class-based majority vote consensus models are more effective in meeting these criteria for all models under investigation.

**Fig. 5:**
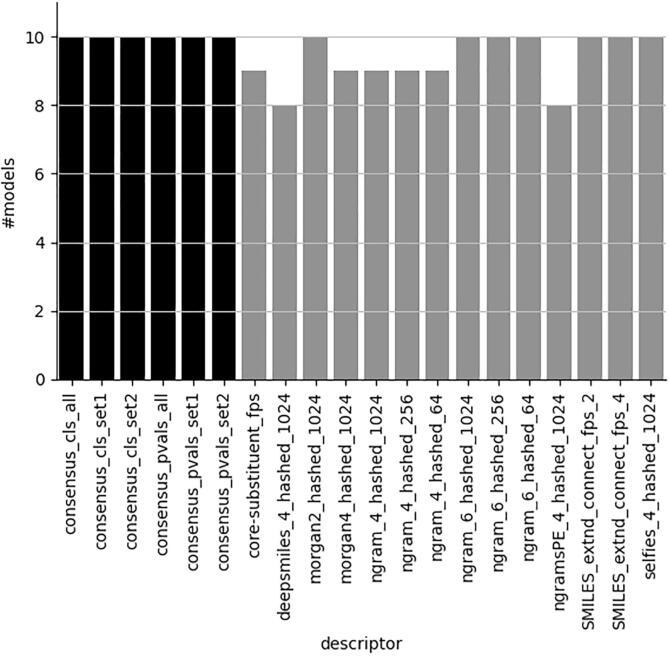
Number of valid models at significance level 0.2 regardless of level of efficiency. Maximum number is 10. Consensus models in black. See Fig. 1 for an explanation of descriptor and concensus models nomenclature.

Other types of single descriptor set models, although valid for the most part, as shown in Fig. 5, do not reach an efficiency of at least 80 % for both classes at this significance level (Fig. 4 right panel). Lowering the efficiency level to 0.75 (Fig. 4 left panel) improves the number of acceptable models considerably for the single descriptor set models. However, given the short computational times for generating descriptors, there is little need to compromise with respect to providing a higher level of efficiency. Although both reduced sets of descriptors perform well, the advantage of using set2, containing the N-grams described in subsection “N-grams”, is that these descriptors handle all symbols occurring in SMILES strings. This makes the N-grams very suitable for handling a variety of compounds, including metals and salts, without removing certain parts of the structure, e.g. salts or metals, that may be important for the compounds to exhibit their experimentally measured toxicities.

#### Individual model performance

Section “Overall model performance” described how models based on various descriptor sets, including consensus models, performed across all datasets. This section will discuss which of the above models, with an efficiency of at least 0.75 for both classes, show the best results for each individual dataset.

**Fig. 6:**
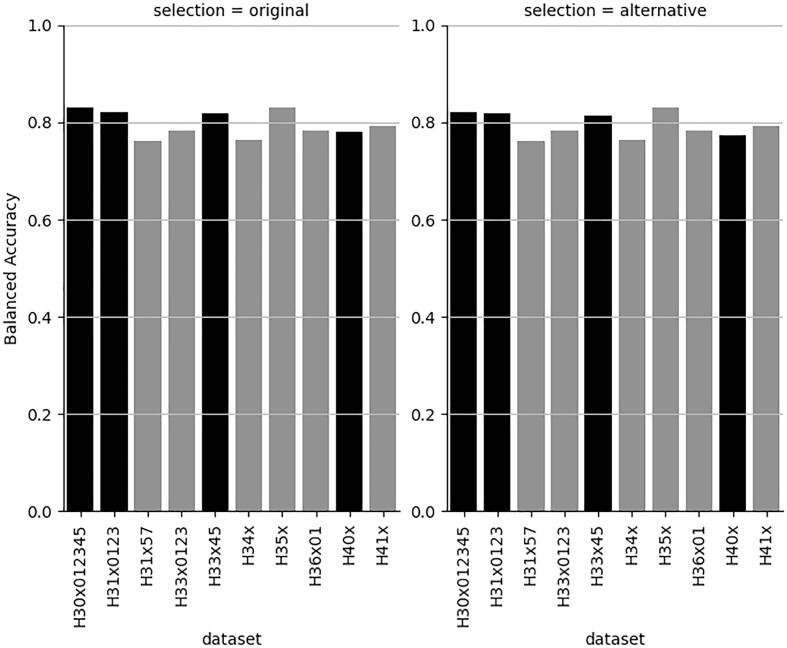
Best balanced accuracy with conformal prediction efficiencies ≥ 0.75 for both classes (left) and with very minor decrease in BA for 4 datasets (right). Black bars indicate the affected datasets.

In order to reach sufficient efficiency (domain coverage) of at least 75 % or 80 % as indicated before in Fig. 1, Fig. 2, respectively, the CP significance level (% errors accepted) needs, for most datasets, to be 0.2 or in some cases 0.15 for individual models. This is manifested in Fig. 6 were all models show balanced accuracies (BAs) of close to 80 %.

**Fig. 7:**
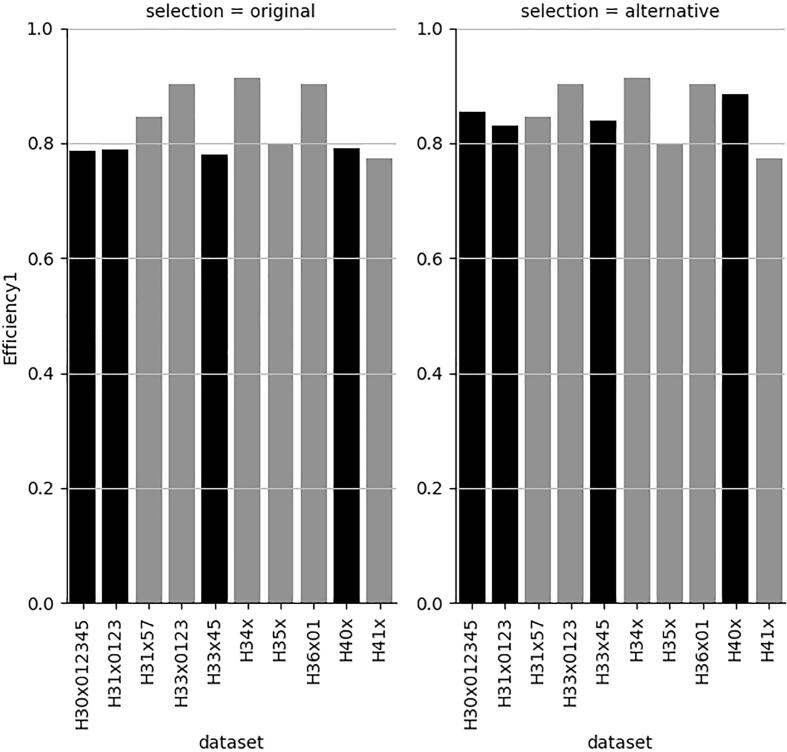
Efficiency for the H-statement labelled class with best BA and with very minor decrease in BA for 4 datasets (right). Black bars indicate the affected datasets.

**Fig. 8:**
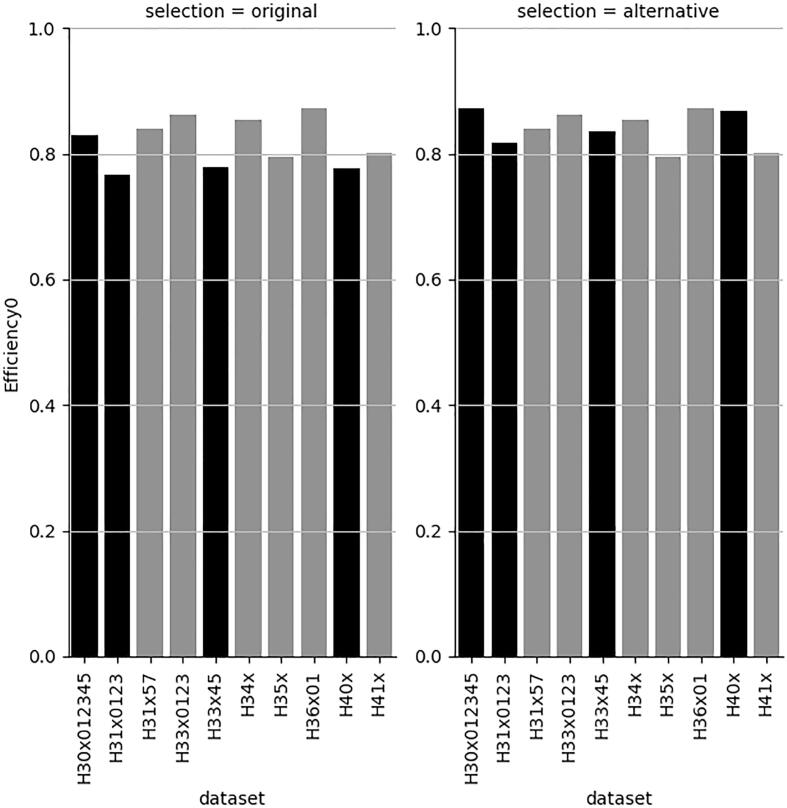
Efficiency for the non-H-statement labelled class with best BA and with very minor decrease in BA for 4 datasets (right). Black bars indicate the affected datasets.

Fig. 6 also shows that a very minor decrease in BA increases the efficiency of some models (Fig. 7, Fig. 8, respectively) above 80 %.

**Fig. 9:**
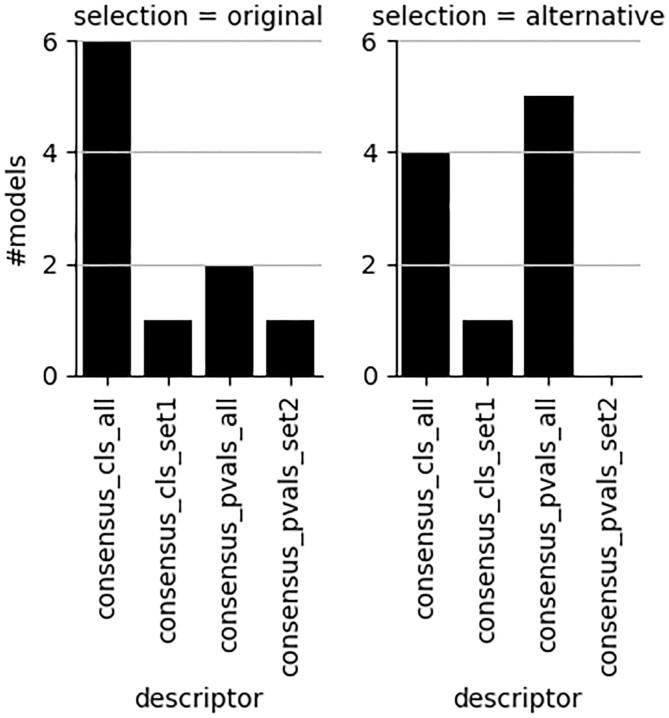
Changes in descriptor sets with best BA (left) and with very minor decrease in BA for 4 datasets (right). See Fig. 1 for an explanation of concensus models nomenclature.

In Fig. 9 it can be noted, as already indicated in Fig. 1, Fig. 2, Fig. 3, Fig. 4, that consensus models show better results in terms of validity, efficiency as well as balanced accuracy. The resulting best individual models in Fig. 6 are all based on some consensus model. Fig. 9 also shows the consequence in terms of descriptor sets when increasing the efficiency by means of a small decrease in BA (Fig. 6). There is shift from majority class determination (consensus_cls_all) to overall median p-value determination of predicted class label (consensus_pvals_all). It is not surprising to see this result since both these consensus models are based on the same underlying individual models and descriptor sets and only differ in the final assignment of the class label.

## Conclusions

This investigation shows the utility of N-grams and other fingerprint featurization procedures for predicting CLP classification, particularly in an ensemble (consensus) setting, where several sets of N-grams and/or fingerprints are selected for the ensemble. Consensus modelling by class or CP median p-values seems to be particularly advantageous in order to obtain both high conformal prediction *validity* but also *efficiency*, e.g. high proportion of single label predictions, as well as good balanced accuracy, sensitivity and specificity. Utilization of the N-grams described in section “N-grams“ allows for handling of all symbols in SMILES strings including those related to metals and salts that may be important for the compounds to exhibit their experimental verified toxicities.

Compared to previous studies that predict chemical toxicity with machine learning and the conformal prediction technique, models developed in this study have good conformal prediction validity and efficiency especially the class based consensus models (Supplementary Table S2 and Fig. S1). The models developed in this study are efficient tools to access hazard classification H-statements of chemicals, which can be useful for chemical hazard assessment, read-across as well as risk management.

### Future Perspectives

While this study has demonstrated the utility of N-grams and consensus modeling for H-statement prediction, several practical steps can be taken to further refine and expand this approach. First, there is potential to integrate more advanced machine learning techniques, such as deep learning and graph neural networks, which could capture more complex molecular features. While these methods are not essential, they offer the opportunity to further enhance predictive accuracy, particularly for more challenging or nuanced toxicity endpoints.

Second, a critical aspect of model validation and regulatory acceptance is experimental verification of the predicted classifications. While the models developed in this study provide statistically robust predictions for the data extracted from the REACH dossiers, experimental data from other sources could be further employed to validate the hazard classifications. For example, in vitro assays, such as cytotoxicity screening, Ames tests for mutagenicity, or OECD-guideline-based toxicological assays, could serve as benchmarks for comparison with model predictions. High-throughput screening methods, such as ToxCast and Tox21 bioassays, could also be leveraged to validate the model’s ability to predict specific hazard endpoints. Additionally, in silico-to-in vivo extrapolations could be evaluated by comparing predictions with existing animal toxicity data where available, ensuring that the models align with observed toxicological effects.

Third, a valuable direction for future work is to compare the performance of the models developed in this study with previously published models that were trained on different datasets for the same or similar endpoints, as publicly available prediction models for H-statements classification are limited. When comparing with previous studies that predicts relevant toxicity endpoints, it worth to be noted that the endpoint definition and test guidlines are usually different. Even so, conducting a thorough benchmarking analysis could provide insights into how the REACH CLP-based models perform relative to models trained on public datasets, and highlight the strengths and limitations of using regulatory datasets. Also, as different data source lead to different coverage of chemical space, consensus models can be developed by combining both models trained on regulatory datasets and other public datasets to have broader applicability domains and better prediction reliabilities.

Furthermore, exploring the potential of combining the REACH database used in this study with additional European regulatory or literature databases could expand the chemical space and increase the diversity of the data. Integrating complementary datasets, such as other ECHA- managed resources or OECD chemical safety databases, could provide a more comprehensive view of chemical hazards and improve the model’s generalizability.

Finally, to align these in silico models with regulatory frameworks, specific steps should be taken to ensure their applicability in CLP hazard classification. First, collaborating with European regulatory bodies and applying the models in European research projects could enable validation of model prediction against officially classified substances. Second, engagement of stakeholders—including industry representatives and policymakers—would help integrate the models into workflows of chemical risk assessment, life cycle assessment, or safe and sustainable by design (SSbD). Also, developing standardized reporting formats aligned with regulatory guidelines could facilitate the adoption of these models in hazard classification. By systematically addressing these aspects, these computational approaches could become practical tools for supporting regulatory decision-making, reducing reliance on animal testing, and enhancing chemical safety assessments.

**Code availability**

The code used in this work is available at the various repositories mentioned in section “ Machine Learning methods and featurization” and the GitHub repository: https://github.com/ulfn1/hphrase.

## CRediT authorship contribution statement

**Ulf Norinder:** Conceptualization, Methodology, Formal analysis, Software, Validation, Visualization, Investigation, Writing – original draft. **Ziye Zheng:** Conceptualization, Data curation, Methodology, Formal analysis, Software, Validation, Visualization, Investigation, Writing – review & editing. **Ian Cotgreave:** Supervision, Funding acquisition, Writing – review & editing.

## Declaration of competing interest

The authors declare that they have no known competing financial interests or personal relationships that could have appeared to influence the work reported in this paper.

## Data Availability

The data cannot be made available by the authors due to ECHA regulations. However, the data can be retrieved using the procedure outlined in section “Data retrieval” and Supplementary Material (pseudo code).
