# Supplementary material for: Prediction of the classification, labelling and packaging regulation H-statements with confidence using conformal prediction with N-grams and molecular fingerprints
Source: Curr Res Toxicol. 2025 May 22;8:100242. doi: 10.1016/j.crtox.2025.100242 (PMC12163164; doi:10.1016/j.crtox.2025.100242)
Supplement: Supplementary Data 1 [file mmc1.docx]

**Supplementary Material**

Prediction of the classification, labelling and packaging Regulation H-statements with confidence using Conformal Prediction with N-grams and Molecular Fingerprints

Ulf Norinder, Ziye Zheng, and Ian Cotgreave

**Table S1. H-statement groups and included H-statements and number compounds in each class**.

| **H-statement group** | **Included H-statements** | **Corresponding endpoints** | **#cmpds H-statement class** | **#cmpds non-H-statement class** |
| --- | --- | --- | --- | --- |
| H30x012345 | H300, H301, H302, H303, H304. H305 | Fatal or harmful if swallowed | 4481 | 3108 |
| H31x0123 | H310, H311, H312, H313 | Fatal or harmful in contact with skin | 1067 | 5942 |
| H31x57 | H315, H317 | Skin irritation | 4931 | 5342 |
| H33x0123 | H330, H331, H332, H333 | Fatal or harmful if inhaled | 393 | 8433 |
| H33x45 | H334, H335 | Respiratory irritation | 1708 | 1994 |
| H34x | All subcategories of H34 | Genetic toxicity | 393 | 8433 |
| H35x | All subcategories of H35 | Carcinogenicity | 510 | 2769 |
| H36x01 | H360x,H361x | May damage fertility or unborn child | 902 | 4894 |
| H40x | All subcategories of H40 | Toxic to aquatic life | 1754 | 7615 |
| H41x | All subcategories of H41 | Toxic to aquatic life with long lasting effects | 4847 | 5040 |

**Table S2. Comparison of H-statement models with some other published Conformal Prediction models for toxicity endpoints (validity and efficiency).^#^**

| **Dataset** | **Method** | **Significance level** | **validity1** | **validity0** | **efficiency1** | **efficiency0** | **#datasets** | **model_type** | **reference** |
| --- | --- | --- | --- | --- | --- | --- | --- | --- | --- |
| PubChem datasets | RF_rdkit | 0.2 | 0.833 | 0.824 | 0.867 | 0.835 | 16 | single_model | S1 |
| H-statements | consensus_cls_all | 0.2 | 0.808 | 0.79 | 0.916 | 0.899 | 10 | consensus | this work |
| H-statements | consensus_cls_set1 | 0.2 | 0.81 | 0.788 | 0.9 | 0.889 | 10 | consensus | this work |
| H-statements | consensus_cls_set2 | 0.2 | 0.801 | 0.786 | 0.887 | 0.87 | 10 | consensus | this work |
| H-statements | consensus_pvals_all | 0.2 | 0.857 | 0.852 | 0.786 | 0.771 | 10 | consensus | this work |
| H-statements | consensus_pvals_set1 | 0.2 | 0.851 | 0.846 | 0.779 | 0.775 | 10 | consensus | this work |
| H-statements | consensus_pvals_set2 | 0.2 | 0.832 | 0.826 | 0.802 | 0.789 | 10 | consensus | this work |
| H-statements | core-substituent_fps | 0.2 | 0.804 | 0.798 | 0.763 | 0.771 | 10 | single_model | this work |
| H-statements | deepsmiles_4_hashed_1024 | 0.2 | 0.802 | 0.802 | 0.807 | 0.811 | 10 | single_model | this work |
| H-statements | morgan2_hashed_1024 | 0.2 | 0.799 | 0.798 | 0.853 | 0.841 | 10 | single_model | this work |
| H-statements | morgan4_hashed_1024 | 0.2 | 0.799 | 0.801 | 0.836 | 0.818 | 10 | single_model | this work |
| H-statements | ngram_4_hashed_1024 | 0.2 | 0.799 | 0.798 | 0.847 | 0.839 | 10 | single_model | this work |
| H-statements | ngram_4_hashed_256 | 0.2 | 0.802 | 0.796 | 0.826 | 0.808 | 10 | single_model | this work |
| H-statements | ngram_4_hashed_64 | 0.2 | 0.803 | 0.803 | 0.811 | 0.795 | 10 | single_model | this work |
| H-statements | ngram_6_hashed_1024 | 0.2 | 0.802 | 0.801 | 0.825 | 0.806 | 10 | single_model | this work |
| H-statements | ngram_6_hashed_256 | 0.2 | 0.8 | 0.797 | 0.807 | 0.802 | 10 | single_model | this work |
| H-statements | ngram_6_hashed_64 | 0.2 | 0.798 | 0.799 | 0.797 | 0.784 | 10 | single_model | this work |
| H-statements | ngramsPE_4_hashed_1024 | 0.2 | 0.799 | 0.8 | 0.809 | 0.813 | 10 | single_model | this work |
| H-statements | SMILES_extnd_connect_fps_2 | 0.2 | 0.8 | 0.802 | 0.825 | 0.818 | 10 | single_model | this work |
| H-statements | SMILES_extnd_connect_fps_4 | 0.2 | 0.799 | 0.801 | 0.827 | 0.818 | 10 | single_model | this work |
| H-statements | selfies_4_hashed_1024 | 0.2 | 0.806 | 0.807 | 0.771 | 0.765 | 10 | single_model | this work |
| tox21 | LGBM_rdkit | 0.2 | 0.795 | 0.798 | 0.933 | 0.897 | 12 | single_model | S2 |
| tox21 | DNN8_morgan4 | 0.2 | 0.799 | 0.8 | 0.896 | 0.837 | 12 | single_model | S2 |
| tox21 | DNN4_morgan4 | 0.2 | 0.793 | 0.801 | 0.914 | 0.839 | 12 | single_model | S2 |
| tox21 | LGBM_morgan4 | 0.2 | 0.792 | 0.8 | 0.917 | 0.861 | 12 | single_model | S2 |
| tox21 | DNN8_rdkit | 0.2 | 0.796 | 0.798 | 0.889 | 0.863 | 12 | single_model | S2 |
| tox21 | DNN4_rdkit | 0.2 | 0.793 | 0.798 | 0.91 | 0.87 | 12 | single_model | S2 |
| tox21 | RF_rdkit | 0.2 | 0.806 | 0.801 | 0.929 | 0.895 | 12 | single_model | S2 |
| tox21 | RF_morgan4 | 0.2 | 0.796 | 0.8 | 0.931 | 0.899 | 12 | single_model | S2 |
| catmos_nt | RF_rdkit | 0.2 | 0.8 | 0.805 | 0.909 | 0.912 | 1 | single_model | S3 |
| catmos_nt | RF_cddd | 0.2 | 0.802 | 0.824 | 0.855 | 0.879 | 1 | single_model | S3 |
| catmos_nt | mg_bert | 0.2 | 0.798 | 0.848 | 0.814 | 0.845 | 1 | single_model | S3 |
| catmos_nt | molbert_p | 0.2 | 0.791 | 0.83 | 0.856 | 0.864 | 1 | single_model | S3 |
| catmos_nt | molbert | 0.2 | 0.797 | 0.83 | 0.873 | 0.892 | 1 | single_model | S3 |
| catmos_vt | RF_rdkit | 0.2 | 0.819 | 0.821 | 0.996 | 0.979 | 1 | single_model | S3 |
| catmos_vt | mg_bert | 0.2 | 0.843 | 0.829 | 0.923 | 0.9 | 1 | single_model | S3 |
| catmos_vt | molbert_p | 0.2 | 0.798 | 0.819 | 0.996 | 0.991 | 1 | single_model | S3 |
| catmos_vt | molbert | 0.2 | 0.815 | 0.818 | 0.944 | 0.931 | 1 | single_model | S3 |
| catmos_vt | RF_cddd | 0.2 | 0.77 | 0.817 |  |  | 1 | single_model | S3 |
| ames mutagenicity | RF_LS_fingerprints_internal | 0.2 | 0.803 | 0.797 | 0.824 | | 1 | overall_efficiency | S4 |
| ames mutagenicity | RF_LS_fingerprints_external | 0.2 | 0.794 | 0.818 | 0.861 | | 1 | overall_efficiency | S4 |
| ames mutagenicity | RF_LS_PAA_feature_internal | 0.2 | 0.802 | 0.795 | 0.908 | | 1 | overall_efficiency | S4 |
| ames mutagenicity | RF_LS_PAA_feature_external | 0.2 | 0.81 | 0.802 | 0.937 | | 1 | overall_efficiency | S4 |
| AID1851 | SVM_rdkit | 0.2 | 0.829 | | 0.909 | | 1 | overall_efficiency | S5 |
| AID2796 | SVM_rdkit | 0.2 | 0.853 | | 0.901 | | 1 | overall_efficiency | S5 |
| AID493091 | SVM_rdkit | 0.2 | 0.79 | | 0.908 | | 1 | overall_efficiency | S5 |
| ames mutagenicity | SVM_rdkit | 0.2 | 0.852 | | 0.829 | | 1 | overall_efficiency | S5 |
| MNT | CHEM | 0.2 | 0.77 | | 0.76 | | 1 | overall_efficiency | S6 |
| MNT | BIO | 0.2 | 0.82 | | 0.81 | | 1 | overall_efficiency | S6 |
| MNT | CHEMBIO | 0.2 | 0.81 | | 0.85 | | 1 | overall_efficiency | S6 |
| DILI | CHEM | 0.2 | 0.78 | | 0.91 | | 1 | overall_efficiency | S6 |
| DILI | BIO | 0.2 | 0.81 | | 0.83 | | 1 | overall_efficiency | S6 |
| DILI | CHEMBIO | 0.2 | 0.81 | | 0.88 | | 1 | overall_efficiency | S6 |
| DICC | CHEM | 0.2 | 0.79 | | 0.84 | | 1 | overall_efficiency | S6 |
| DICC | BIO | 0.2 | 0.79 | | 0.96 | | 1 | overall_efficiency | S6 |
| DICC | CHEMBIO | 0.2 | 0.79 | | 0.94 | | 1 | overall_efficiency | S6 |

# validity1 = validity for the "active" class, validity0 = validity for the "inactive” class, efficiency1 = efficiency for the "active" class, efficiency0 = efficiency for the "inactive” class

**References**

S1. Svensson F, Norinder U, Bender A. Modelling compound cytotoxicity using conformal prediction and PubChem HTS data. Toxicol Res (Camb). 2016;6(1):73-80. doi: 10.1039/c6tx00252h.

S2. Zhang J, Norinder U, Svensson F. Deep Learning-Based Conformal Prediction of Toxicity. J Chem Inf Model. 2021;61(6):2648-2657. doi: 10.1021/acs.jcim.1c00208.

S3. Norinder U. Traditional Machine and Deep Learning for Predicting Toxicity Endpoints. Molecules. 2022;28(1):217. doi: 10.3390/molecules28010217.

S4. Norinder U, Myatt G, Ahlberg E. Predicting Aromatic Amine Mutagenicity with Confidence: A Case Study Using Conformal Prediction. Biomolecules. 2018;8(3):85. doi: 10.3390/biom8030085.

S5. Norinder U, Boyer S. Binary classification of imbalanced datasets using conformal prediction. J Mol Graph Model. 2017;72:256-265. doi: 10.1016/j.jmgm.2017.01.008.

S6. Garcia de Lomana M, Morger A, Norinder U, Buesen R, Landsiedel R, Volkamer A, Kirchmair J, Mathea M. ChemBioSim: Enhancing Conformal Prediction of In Vivo Toxicity by Use of Predicted Bioactivities. J Chem Inf Model. 2021;61(7):3255-3272. doi: 10.1021/acs.jcim.1c00451.

**Figure S1. Comparison of H-statement models with some other published Conformal Prediction models for toxicity endpoints (validity and efficiency).^#^**

**
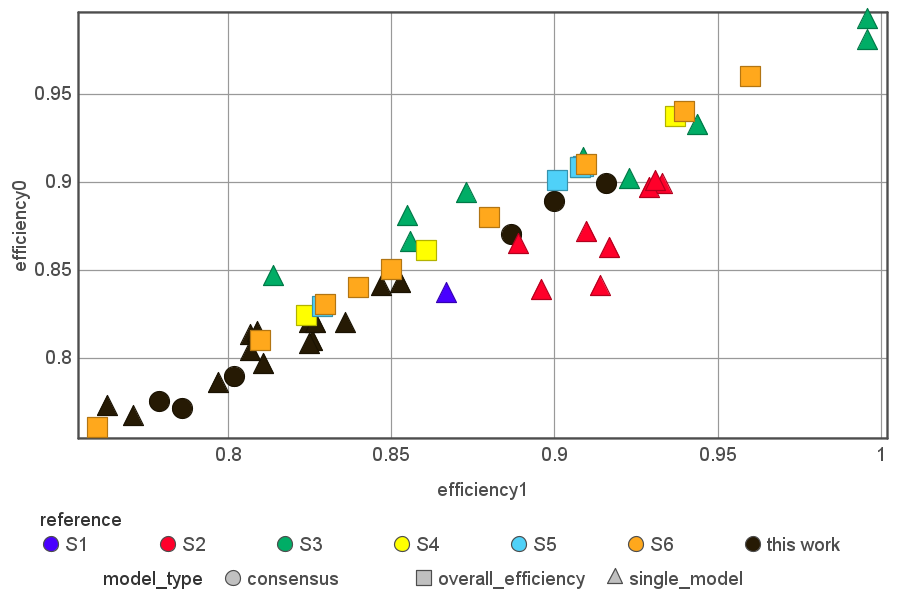
**

# efficiency1 = efficiency for the "active" class, efficiency0 = efficiency for the "inactive” class

**Pseudo code for retrieving H-statements**

1. IMPORT python libraries BeautifulSoup and pandas

2. Initialize an empty pandas dataFrame: df

2.1 id_list = ["Display Name", "EC Number", "EC Name", "CAS Number", "Molecular formula", "IUPAC Name"]

2.2 type_list = ["Composition", "Origin"]

2.2 admin_list = ["Total range", "Registered as", "Submitted"]

2.3 human_health_classes = ["Acute toxicity - oral", "Acute toxicity - dermal", "Acute toxicity - inhalation", "Skin corrosion / irritation", "Serious eye damage / eye irritation", "Respiratory sensitisation", "Skin sensitisation", "Aspiration hazard", "Reproductive toxicity", "Effects on or via lactation", "Germ cell mutagenicity", "Carcinogenicity", "1. Specific target organ toxicity - single (STOT-SE)", "1. Specific target organ toxicity - repeated (STOT-RE)"]

2.4 ecotox_classes = ["Hazardous to the aquatic environment (acute / short-term)", "Hazardous to the aquatic environment (long-term)", "Hazardous to the ozone layer"]

2.5 columns of df = "id"+id_list+type_list+admin_list+human_health_list+ecotox_list

3. Define the function "fetch_data_for_id(i)" to fetch data for a given id i

3.1 Create a row in the DataFrame for the current id: df[i, "id"] = i

3.2 Initialize the URLs for i

3.2.1 url0 = "https://echa.europa.eu/registration-dossier/-/registered-dossier/i/1/1"

3.2.2 url1 = "https://echa.europa.eu/registration-dossier/-/registered-dossier/i/1/2"

3.2.3 url2 = "https://echa.europa.eu/registration-dossier/-/registered-dossier/i/2/1"

3.3 Make requests to the URLs and parse the HTML content to get information into df with BeautifulSoup:

3.3.1 columns in id_list can be fetched from url0 under <h3> title "sIdentification"

3.3.2 columns in type_list can be fetched from url0 under <h3> title "sTypeOfSubstance"

3.3.3 columns in admin_list can be fetched from url1 in a <div> object with id "SectionContent"

3.3.4 columns in human_health_classes can be fetched from url2 in a <div> object with id "collapseHealthHazards1CollapseGroup1"

3.3.5 columns in ecotox_classes can be fetched from url2 in a <div> object with id "collapseEnvironmentalHazards1CollapseGroup1"

Example (get df.loc[i, "Display Name"]):

html_list = list of all html objects in url0 fetched by BeautifulSoup

for a in the range from 0 to the length of html_list:

if html_list[a] is a <h3> object named "sIdentification" and html_list[a+1] is a <dl> object:

fetched_id_list = html_list[a+1]

for b in range from 0 to the length of fetched_id_list:

if id_list[b] is a <dt> object with the value "Display Name" and id_list[b+1] is a <dd> object:

df.loc[i, "Display Name"] = id_list[b+1]

4. Execute the function "fetch_data_for_id(i)" for i ranging from 0 to 40000

5. Save df to csv file with pandas

**H-statements_statistics**

| **Dataset** | **Descriptor** | **Significance level** | **Balanced accuracy** | **Accuracy** | **Sensitivity** | **Specificity** | **MCC** | **ROC_auc^a^** |
| --- | --- | --- | --- | --- | --- | --- | --- | --- |
| H30x012345 | consensus_cls_all | 0.1 | 0.8688 | 0.8684 | 0.8654 | 0.8723 | 0.7349 | NA |
| H30x012345 | consensus_cls_all | 0.15 | 0.8222 | 0.8228 | 0.826 | 0.8183 | 0.6399 | NA |
| H30x012345 | consensus_cls_all | 0.2 | 0.7902 | 0.791 | 0.7948 | 0.7855 | 0.5747 | NA |
| H30x012345 | consensus_cls_all | 0.25 | 0.7836 | 0.7864 | 0.7992 | 0.768 | 0.5629 | NA |
| H30x012345 | consensus_cls_all | 0.3 | 0.7857 | 0.7883 | 0.8 | 0.7714 | 0.5672 | NA |
| H30x012345 | consensus_pvals_all | 0.1 | 0.8839 | 0.8837 | 0.8816 | 0.8862 | 0.7656 | 0.9210 |
| H30x012345 | consensus_pvals_all | 0.15 | 0.8503 | 0.8508 | 0.8536 | 0.847 | 0.6971 | 0.9094 |
| H30x012345 | consensus_pvals_all | 0.2 | 0.8178 | 0.8183 | 0.8205 | 0.8151 | 0.6306 | 0.8990 |
| H30x012345 | consensus_pvals_all | 0.25 | 0.7853 | 0.7863 | 0.7909 | 0.7797 | 0.5649 | 0.8894 |
| H30x012345 | consensus_pvals_all | 0.3 | 0.8076 | 0.8094 | 0.8179 | 0.7974 | 0.6111 | 0.8862 |
| H30x012345 | core-substituent_fps | 0.1 | 0.8236 | 0.8235 | 0.823 | 0.8242 | 0.6428 | 0.8624 |
| H30x012345 | core-substituent_fps | 0.15 | 0.7906 | 0.7905 | 0.7902 | 0.791 | 0.575 | 0.8523 |
| H30x012345 | core-substituent_fps | 0.2 | 0.7633 | 0.7634 | 0.7636 | 0.7631 | 0.5207 | 0.8424 |
| H30x012345 | core-substituent_fps | 0.25 | 0.7375 | 0.7374 | 0.7366 | 0.7385 | 0.469 | 0.8331 |
| H30x012345 | core-substituent_fps | 0.3 | 0.7483 | 0.7485 | 0.749 | 0.7477 | 0.4907 | 0.8291 |
| H30x012345 | deepsmiles_4_hashed_1024 | 0.1 | 0.8309 | 0.8298 | 0.8228 | 0.839 | 0.6579 | 0.8760 |
| H30x012345 | deepsmiles_4_hashed_1024 | 0.15 | 0.7992 | 0.7989 | 0.7973 | 0.801 | 0.5932 | 0.8651 |
| H30x012345 | deepsmiles_4_hashed_1024 | 0.2 | 0.7662 | 0.766 | 0.7648 | 0.7676 | 0.5267 | 0.8547 |
| H30x012345 | deepsmiles_4_hashed_1024 | 0.25 | 0.7419 | 0.7425 | 0.7454 | 0.7384 | 0.478 | 0.8455 |
| H30x012345 | deepsmiles_4_hashed_1024 | 0.3 | 0.7572 | 0.7583 | 0.7634 | 0.751 | 0.5089 | 0.8421 |
| H30x012345 | morgan2_hashed_1024 | 0.1 | 0.8367 | 0.8349 | 0.8229 | 0.8505 | 0.6689 | 0.8892 |
| H30x012345 | morgan2_hashed_1024 | 0.15 | 0.8076 | 0.8065 | 0.8011 | 0.814 | 0.6094 | 0.8775 |
| H30x012345 | morgan2_hashed_1024 | 0.2 | 0.781 | 0.7807 | 0.779 | 0.7831 | 0.5559 | 0.8678 |
| H30x012345 | morgan2_hashed_1024 | 0.25 | 0.769 | 0.7693 | 0.7705 | 0.7675 | 0.5318 | 0.8613 |
| H30x012345 | morgan2_hashed_1024 | 0.3 | 0.7896 | 0.7908 | 0.7966 | 0.7826 | 0.5743 | 0.8601 |
| H30x012345 | morgan4_hashed_1024 | 0.1 | 0.8361 | 0.8344 | 0.8195 | 0.8528 | 0.669 | 0.8774 |
| H30x012345 | morgan4_hashed_1024 | 0.15 | 0.8039 | 0.8031 | 0.7989 | 0.8089 | 0.6031 | 0.8675 |
| H30x012345 | morgan4_hashed_1024 | 0.2 | 0.7764 | 0.7761 | 0.7749 | 0.7779 | 0.5472 | 0.8584 |
| H30x012345 | morgan4_hashed_1024 | 0.25 | 0.7608 | 0.7608 | 0.7608 | 0.7609 | 0.5155 | 0.8510 |
| H30x012345 | morgan4_hashed_1024 | 0.3 | 0.7806 | 0.7821 | 0.7891 | 0.7721 | 0.5567 | 0.8491 |
| H30x012345 | ngram_4_hashed_1024 | 0.1 | 0.8354 | 0.8346 | 0.8287 | 0.8422 | 0.667 | 0.8835 |
| H30x012345 | ngram_4_hashed_1024 | 0.15 | 0.8056 | 0.8051 | 0.8024 | 0.8088 | 0.6058 | 0.8721 |
| H30x012345 | ngram_4_hashed_1024 | 0.2 | 0.7748 | 0.7748 | 0.7746 | 0.7751 | 0.5441 | 0.8623 |
| H30x012345 | ngram_4_hashed_1024 | 0.25 | 0.749 | 0.7499 | 0.7538 | 0.7442 | 0.4923 | 0.8531 |
| H30x012345 | ngram_4_hashed_1024 | 0.3 | 0.7671 | 0.768 | 0.7724 | 0.7617 | 0.5289 | 0.8500 |
| H30x012345 | ngram_4_hashed_256 | 0.1 | 0.8375 | 0.8363 | 0.8277 | 0.8472 | 0.6715 | 0.8848 |
| H30x012345 | ngram_4_hashed_256 | 0.15 | 0.7986 | 0.7972 | 0.7896 | 0.8075 | 0.5916 | 0.8731 |
| H30x012345 | ngram_4_hashed_256 | 0.2 | 0.7673 | 0.7658 | 0.7588 | 0.7758 | 0.5279 | 0.8623 |
| H30x012345 | ngram_4_hashed_256 | 0.25 | 0.7458 | 0.7458 | 0.7456 | 0.7461 | 0.4854 | 0.8526 |
| H30x012345 | ngram_4_hashed_256 | 0.3 | 0.7633 | 0.7639 | 0.7668 | 0.7598 | 0.5211 | 0.8496 |
| H30x012345 | ngram_4_hashed_64 | 0.1 | 0.8406 | 0.8403 | 0.8384 | 0.8428 | 0.6775 | 0.8792 |
| H30x012345 | ngram_4_hashed_64 | 0.15 | 0.8061 | 0.8056 | 0.8031 | 0.809 | 0.6063 | 0.8687 |
| H30x012345 | ngram_4_hashed_64 | 0.2 | 0.774 | 0.774 | 0.7743 | 0.7736 | 0.5418 | 0.8589 |
| H30x012345 | ngram_4_hashed_64 | 0.25 | 0.7473 | 0.7476 | 0.749 | 0.7456 | 0.4885 | 0.8501 |
| H30x012345 | ngram_4_hashed_64 | 0.3 | 0.7641 | 0.7653 | 0.7708 | 0.7575 | 0.523 | 0.8465 |
| H30x012345 | ngram_6_hashed_1024 | 0.1 | 0.8243 | 0.8226 | 0.8098 | 0.8387 | 0.645 | 0.8707 |
| H30x012345 | ngram_6_hashed_1024 | 0.15 | 0.7951 | 0.7937 | 0.7851 | 0.8052 | 0.5855 | 0.8597 |
| H30x012345 | ngram_6_hashed_1024 | 0.2 | 0.7666 | 0.7646 | 0.7548 | 0.7785 | 0.5268 | 0.8499 |
| H30x012345 | ngram_6_hashed_1024 | 0.25 | 0.7451 | 0.7436 | 0.737 | 0.7532 | 0.4833 | 0.8413 |
| H30x012345 | ngram_6_hashed_1024 | 0.3 | 0.7625 | 0.7625 | 0.7625 | 0.7625 | 0.5192 | 0.8385 |
| H30x012345 | ngram_6_hashed_256 | 0.1 | 0.8221 | 0.8197 | 0.7993 | 0.8448 | 0.6407 | 0.8684 |
| H30x012345 | ngram_6_hashed_256 | 0.15 | 0.7935 | 0.7914 | 0.7797 | 0.8072 | 0.5813 | 0.8579 |
| H30x012345 | ngram_6_hashed_256 | 0.2 | 0.7652 | 0.7634 | 0.7553 | 0.775 | 0.5236 | 0.8482 |
| H30x012345 | ngram_6_hashed_256 | 0.25 | 0.7399 | 0.7384 | 0.7317 | 0.7481 | 0.4731 | 0.8392 |
| H30x012345 | ngram_6_hashed_256 | 0.3 | 0.749 | 0.7484 | 0.7454 | 0.7527 | 0.4915 | 0.8353 |
| H30x012345 | ngram_6_hashed_64 | 0.1 | 0.8268 | 0.8258 | 0.8208 | 0.8328 | 0.6482 | 0.8699 |
| H30x012345 | ngram_6_hashed_64 | 0.15 | 0.7915 | 0.7904 | 0.7849 | 0.7981 | 0.5769 | 0.8587 |
| H30x012345 | ngram_6_hashed_64 | 0.2 | 0.7635 | 0.7622 | 0.7564 | 0.7706 | 0.5204 | 0.8485 |
| H30x012345 | ngram_6_hashed_64 | 0.25 | 0.7392 | 0.7375 | 0.7301 | 0.7482 | 0.4716 | 0.8393 |
| H30x012345 | ngram_6_hashed_64 | 0.3 | 0.7561 | 0.7547 | 0.7485 | 0.7637 | 0.5055 | 0.8358 |
| H30x012345 | ngramsPE_4_hashed_1024 | 0.1 | 0.8287 | 0.8271 | 0.8131 | 0.8443 | 0.6545 | 0.8736 |
| H30x012345 | ngramsPE_4_hashed_1024 | 0.15 | 0.798 | 0.7966 | 0.7877 | 0.8083 | 0.5914 | 0.8629 |
| H30x012345 | ngramsPE_4_hashed_1024 | 0.2 | 0.7707 | 0.7692 | 0.762 | 0.7794 | 0.5352 | 0.8523 |
| H30x012345 | ngramsPE_4_hashed_1024 | 0.25 | 0.7475 | 0.7473 | 0.746 | 0.7491 | 0.4888 | 0.8432 |
| H30x012345 | ngramsPE_4_hashed_1024 | 0.3 | 0.7629 | 0.7633 | 0.7649 | 0.7609 | 0.52 | 0.8397 |
| H30x012345 | SMILES_extnd_connect_fps_2_1024 | 0.1 | 0.8424 | 0.8423 | 0.8419 | 0.8428 | 0.6794 | 0.8753 |
| H30x012345 | SMILES_extnd_connect_fps_2_1024 | 0.15 | 0.809 | 0.8091 | 0.81 | 0.808 | 0.6122 | 0.8667 |
| H30x012345 | SMILES_extnd_connect_fps_2_1024 | 0.2 | 0.7781 | 0.7775 | 0.7746 | 0.7816 | 0.5496 | 0.8586 |
| H30x012345 | SMILES_extnd_connect_fps_2_1024 | 0.25 | 0.7555 | 0.7558 | 0.7568 | 0.7542 | 0.5052 | 0.8509 |
| H30x012345 | SMILES_extnd_connect_fps_2_1024 | 0.3 | 0.776 | 0.7765 | 0.7787 | 0.7733 | 0.5462 | 0.8486 |
| H30x012345 | SMILES_extnd_connect_fps_4_1024 | 0.1 | 0.8377 | 0.8371 | 0.8333 | 0.8421 | 0.6708 | 0.8806 |
| H30x012345 | SMILES_extnd_connect_fps_4_1024 | 0.15 | 0.8049 | 0.8048 | 0.8039 | 0.8059 | 0.6047 | 0.8701 |
| H30x012345 | SMILES_extnd_connect_fps_4_1024 | 0.2 | 0.7782 | 0.7785 | 0.7803 | 0.776 | 0.5506 | 0.8609 |
| H30x012345 | SMILES_extnd_connect_fps_4_1024 | 0.25 | 0.7598 | 0.7596 | 0.7586 | 0.761 | 0.5134 | 0.8535 |
| H30x012345 | SMILES_extnd_connect_fps_4_1024 | 0.3 | 0.786 | 0.7875 | 0.7944 | 0.7777 | 0.5673 | 0.8520 |
| H30x012345 | selfies_4_hashed_1024 | 0.1 | 0.8269 | 0.8257 | 0.8164 | 0.8374 | 0.6504 | 0.8651 |
| H30x012345 | selfies_4_hashed_1024 | 0.15 | 0.7951 | 0.7951 | 0.7949 | 0.7954 | 0.5858 | 0.8547 |
| H30x012345 | selfies_4_hashed_1024 | 0.2 | 0.7699 | 0.7699 | 0.7701 | 0.7697 | 0.5342 | 0.8453 |
| H30x012345 | selfies_4_hashed_1024 | 0.25 | 0.7442 | 0.7447 | 0.7473 | 0.7411 | 0.4827 | 0.8366 |
| H30x012345 | selfies_4_hashed_1024 | 0.3 | 0.7506 | 0.7523 | 0.7603 | 0.7408 | 0.4962 | 0.8323 |
| H31x0123 | consensus_cls_all | 0.1 | 0.8667 | 0.8617 | 0.8744 | 0.859 | 0.6257 | NA |
| H31x0123 | consensus_cls_all | 0.15 | 0.8256 | 0.817 | 0.8382 | 0.813 | 0.5264 | NA |
| H31x0123 | consensus_cls_all | 0.2 | 0.7871 | 0.7758 | 0.8035 | 0.7707 | 0.4458 | NA |
| H31x0123 | consensus_cls_all | 0.25 | 0.775 | 0.7626 | 0.7929 | 0.7572 | 0.4214 | NA |
| H31x0123 | consensus_cls_all | 0.3 | 0.7775 | 0.7663 | 0.7936 | 0.7613 | 0.4265 | NA |
| H31x0123 | consensus_pvals_all | 0.1 | 0.8886 | 0.8805 | 0.901 | 0.8762 | 0.6695 | 0.9302 |
| H31x0123 | consensus_pvals_all | 0.15 | 0.8528 | 0.8456 | 0.8633 | 0.8422 | 0.5841 | 0.9174 |
| H31x0123 | consensus_pvals_all | 0.2 | 0.8195 | 0.8151 | 0.8259 | 0.8131 | 0.5125 | 0.9062 |
| H31x0123 | consensus_pvals_all | 0.25 | 0.7862 | 0.7813 | 0.7932 | 0.7791 | 0.4463 | 0.8952 |
| H31x0123 | consensus_pvals_all | 0.3 | 0.7931 | 0.7919 | 0.7948 | 0.7914 | 0.4624 | 0.8894 |
| H31x0123 | core-substituent_fps | 0.1 | 0.8158 | 0.8041 | 0.8333 | 0.7983 | 0.509 | 0.8556 |
| H31x0123 | core-substituent_fps | 0.15 | 0.7781 | 0.7723 | 0.7868 | 0.7694 | 0.4429 | 0.8438 |
| H31x0123 | core-substituent_fps | 0.2 | 0.7569 | 0.7563 | 0.7578 | 0.756 | 0.4021 | 0.8335 |
| H31x0123 | core-substituent_fps | 0.25 | 0.7417 | 0.7396 | 0.7446 | 0.7387 | 0.3699 | 0.8246 |
| H31x0123 | core-substituent_fps | 0.3 | 0.7428 | 0.7451 | 0.7395 | 0.7462 | 0.3725 | 0.8199 |
| H31x0123 | deepsmiles_4_hashed_1024 | 0.1 | 0.8369 | 0.8209 | 0.8609 | 0.813 | 0.5462 | 0.8896 |
| H31x0123 | deepsmiles_4_hashed_1024 | 0.15 | 0.7963 | 0.7852 | 0.8126 | 0.7799 | 0.4682 | 0.8752 |
| H31x0123 | deepsmiles_4_hashed_1024 | 0.2 | 0.7674 | 0.7574 | 0.782 | 0.7528 | 0.412 | 0.8629 |
| H31x0123 | deepsmiles_4_hashed_1024 | 0.25 | 0.7375 | 0.7339 | 0.7427 | 0.7323 | 0.3603 | 0.8511 |
| H31x0123 | deepsmiles_4_hashed_1024 | 0.3 | 0.7361 | 0.7389 | 0.7321 | 0.7401 | 0.359 | 0.8441 |
| H31x0123 | morgan2_hashed_1024 | 0.1 | 0.845 | 0.8384 | 0.8549 | 0.8351 | 0.5734 | 0.8904 |
| H31x0123 | morgan2_hashed_1024 | 0.15 | 0.8063 | 0.8016 | 0.8132 | 0.7994 | 0.4912 | 0.8791 |
| H31x0123 | morgan2_hashed_1024 | 0.2 | 0.7759 | 0.7711 | 0.7829 | 0.7689 | 0.4292 | 0.8692 |
| H31x0123 | morgan2_hashed_1024 | 0.25 | 0.7493 | 0.75 | 0.7483 | 0.7503 | 0.3825 | 0.8601 |
| H31x0123 | morgan2_hashed_1024 | 0.3 | 0.7731 | 0.7726 | 0.774 | 0.7723 | 0.4249 | 0.8575 |
| H31x0123 | morgan4_hashed_1024 | 0.1 | 0.8319 | 0.8289 | 0.8364 | 0.8274 | 0.542 | 0.8822 |
| H31x0123 | morgan4_hashed_1024 | 0.15 | 0.8008 | 0.7976 | 0.8054 | 0.7962 | 0.4782 | 0.8703 |
| H31x0123 | morgan4_hashed_1024 | 0.2 | 0.7706 | 0.7648 | 0.7791 | 0.7622 | 0.4189 | 0.8599 |
| H31x0123 | morgan4_hashed_1024 | 0.25 | 0.7489 | 0.7436 | 0.7565 | 0.7412 | 0.3786 | 0.8507 |
| H31x0123 | morgan4_hashed_1024 | 0.3 | 0.7624 | 0.7595 | 0.7665 | 0.7583 | 0.4026 | 0.8472 |
| H31x0123 | ngram_4_hashed_1024 | 0.1 | 0.8305 | 0.8321 | 0.8281 | 0.8329 | 0.5484 | 0.8704 |
| H31x0123 | ngram_4_hashed_1024 | 0.15 | 0.7971 | 0.794 | 0.8017 | 0.7925 | 0.4731 | 0.8600 |
| H31x0123 | ngram_4_hashed_1024 | 0.2 | 0.7718 | 0.7676 | 0.7778 | 0.7658 | 0.4236 | 0.8507 |
| H31x0123 | ngram_4_hashed_1024 | 0.25 | 0.7414 | 0.7388 | 0.7452 | 0.7377 | 0.3679 | 0.8418 |
| H31x0123 | ngram_4_hashed_1024 | 0.3 | 0.7522 | 0.7485 | 0.7575 | 0.7468 | 0.3861 | 0.8376 |
| H31x0123 | ngram_4_hashed_256 | 0.1 | 0.8259 | 0.8176 | 0.8383 | 0.8135 | 0.5327 | 0.8745 |
| H31x0123 | ngram_4_hashed_256 | 0.15 | 0.7954 | 0.7897 | 0.8038 | 0.787 | 0.4696 | 0.8625 |
| H31x0123 | ngram_4_hashed_256 | 0.2 | 0.7629 | 0.7563 | 0.7724 | 0.7533 | 0.4074 | 0.8519 |
| H31x0123 | ngram_4_hashed_256 | 0.25 | 0.7374 | 0.7347 | 0.7413 | 0.7335 | 0.3634 | 0.8417 |
| H31x0123 | ngram_4_hashed_256 | 0.3 | 0.7399 | 0.7397 | 0.7401 | 0.7397 | 0.3662 | 0.8361 |
| H31x0123 | ngram_4_hashed_64 | 0.1 | 0.823 | 0.8181 | 0.8302 | 0.8158 | 0.5234 | 0.8648 |
| H31x0123 | ngram_4_hashed_64 | 0.15 | 0.7951 | 0.79 | 0.8025 | 0.7877 | 0.4677 | 0.8543 |
| H31x0123 | ngram_4_hashed_64 | 0.2 | 0.7713 | 0.7646 | 0.7809 | 0.7617 | 0.417 | 0.8459 |
| H31x0123 | ngram_4_hashed_64 | 0.25 | 0.7438 | 0.7408 | 0.748 | 0.7395 | 0.3716 | 0.8375 |
| H31x0123 | ngram_4_hashed_64 | 0.3 | 0.7489 | 0.748 | 0.7502 | 0.7476 | 0.3805 | 0.8331 |
| H31x0123 | ngram_6_hashed_1024 | 0.1 | 0.8297 | 0.8234 | 0.8389 | 0.8204 | 0.5384 | 0.8680 |
| H31x0123 | ngram_6_hashed_1024 | 0.15 | 0.7982 | 0.7948 | 0.8032 | 0.7932 | 0.4778 | 0.8568 |
| H31x0123 | ngram_6_hashed_1024 | 0.2 | 0.7686 | 0.7634 | 0.7761 | 0.7611 | 0.4173 | 0.8475 |
| H31x0123 | ngram_6_hashed_1024 | 0.25 | 0.7498 | 0.744 | 0.7583 | 0.7414 | 0.3819 | 0.8390 |
| H31x0123 | ngram_6_hashed_1024 | 0.3 | 0.7491 | 0.7457 | 0.754 | 0.7442 | 0.381 | 0.8342 |
| H31x0123 | ngram_6_hashed_256 | 0.1 | 0.8251 | 0.8224 | 0.8291 | 0.8211 | 0.5296 | 0.8543 |
| H31x0123 | ngram_6_hashed_256 | 0.15 | 0.7946 | 0.7898 | 0.8015 | 0.7877 | 0.465 | 0.8462 |
| H31x0123 | ngram_6_hashed_256 | 0.2 | 0.7682 | 0.7584 | 0.7823 | 0.754 | 0.4118 | 0.8383 |
| H31x0123 | ngram_6_hashed_256 | 0.25 | 0.7408 | 0.7327 | 0.7524 | 0.7292 | 0.3645 | 0.8303 |
| H31x0123 | ngram_6_hashed_256 | 0.3 | 0.7448 | 0.7396 | 0.7522 | 0.7374 | 0.3715 | 0.8260 |
| H31x0123 | ngram_6_hashed_64 | 0.1 | 0.8278 | 0.8114 | 0.8526 | 0.803 | 0.533 | 0.8647 |
| H31x0123 | ngram_6_hashed_64 | 0.15 | 0.7945 | 0.7789 | 0.8177 | 0.7713 | 0.4638 | 0.8551 |
| H31x0123 | ngram_6_hashed_64 | 0.2 | 0.7581 | 0.7484 | 0.7723 | 0.744 | 0.3975 | 0.8456 |
| H31x0123 | ngram_6_hashed_64 | 0.25 | 0.737 | 0.7284 | 0.7495 | 0.7246 | 0.3585 | 0.8359 |
| H31x0123 | ngram_6_hashed_64 | 0.3 | 0.7288 | 0.7219 | 0.7386 | 0.7189 | 0.3449 | 0.8292 |
| H31x0123 | ngramsPE_4_hashed_1024 | 0.1 | 0.8198 | 0.815 | 0.8268 | 0.8127 | 0.5207 | 0.8717 |
| H31x0123 | ngramsPE_4_hashed_1024 | 0.15 | 0.7897 | 0.7817 | 0.8015 | 0.7779 | 0.4604 | 0.8600 |
| H31x0123 | ngramsPE_4_hashed_1024 | 0.2 | 0.7593 | 0.7507 | 0.7718 | 0.7468 | 0.4014 | 0.8490 |
| H31x0123 | ngramsPE_4_hashed_1024 | 0.25 | 0.7324 | 0.7252 | 0.743 | 0.7219 | 0.3521 | 0.8392 |
| H31x0123 | ngramsPE_4_hashed_1024 | 0.3 | 0.7255 | 0.7214 | 0.7313 | 0.7196 | 0.3407 | 0.8322 |
| H31x0123 | SMILES_extnd_connect_fps_2_1024 | 0.1 | 0.8415 | 0.8379 | 0.8467 | 0.8362 | 0.5649 | 0.8744 |
| H31x0123 | SMILES_extnd_connect_fps_2_1024 | 0.15 | 0.8068 | 0.8043 | 0.8105 | 0.8031 | 0.4902 | 0.8656 |
| H31x0123 | SMILES_extnd_connect_fps_2_1024 | 0.2 | 0.779 | 0.7765 | 0.7825 | 0.7754 | 0.4356 | 0.8569 |
| H31x0123 | SMILES_extnd_connect_fps_2_1024 | 0.25 | 0.7511 | 0.7551 | 0.7452 | 0.7569 | 0.388 | 0.8489 |
| H31x0123 | SMILES_extnd_connect_fps_2_1024 | 0.3 | 0.7667 | 0.7696 | 0.7625 | 0.7708 | 0.4169 | 0.8457 |
| H31x0123 | SMILES_extnd_connect_fps_4_1024 | 0.1 | 0.8367 | 0.8326 | 0.8427 | 0.8306 | 0.5536 | 0.8868 |
| H31x0123 | SMILES_extnd_connect_fps_4_1024 | 0.15 | 0.8102 | 0.8068 | 0.8151 | 0.8053 | 0.4921 | 0.8756 |
| H31x0123 | SMILES_extnd_connect_fps_4_1024 | 0.2 | 0.7783 | 0.778 | 0.7787 | 0.7779 | 0.4361 | 0.8649 |
| H31x0123 | SMILES_extnd_connect_fps_4_1024 | 0.25 | 0.7584 | 0.7594 | 0.757 | 0.7598 | 0.3981 | 0.8564 |
| H31x0123 | SMILES_extnd_connect_fps_4_1024 | 0.3 | 0.7768 | 0.7786 | 0.7742 | 0.7794 | 0.4317 | 0.8537 |
| H31x0123 | selfies_4_hashed_1024 | 0.1 | 0.8209 | 0.8196 | 0.8228 | 0.819 | 0.5318 | 0.8666 |
| H31x0123 | selfies_4_hashed_1024 | 0.15 | 0.7835 | 0.7786 | 0.7908 | 0.7762 | 0.4493 | 0.8549 |
| H31x0123 | selfies_4_hashed_1024 | 0.2 | 0.7533 | 0.7521 | 0.755 | 0.7515 | 0.393 | 0.8432 |
| H31x0123 | selfies_4_hashed_1024 | 0.25 | 0.7288 | 0.7261 | 0.7327 | 0.7249 | 0.348 | 0.8327 |
| H31x0123 | selfies_4_hashed_1024 | 0.3 | 0.7209 | 0.7199 | 0.7224 | 0.7194 | 0.334 | 0.8251 |
| H31x57 | consensus_cls_all | 0.1 | 0.8433 | 0.8432 | 0.8452 | 0.8414 | 0.6855 | NA |
| H31x57 | consensus_cls_all | 0.15 | 0.8016 | 0.8014 | 0.8048 | 0.7984 | 0.6027 | NA |
| H31x57 | consensus_cls_all | 0.2 | 0.7653 | 0.765 | 0.7738 | 0.7569 | 0.5303 | NA |
| H31x57 | consensus_cls_all | 0.25 | 0.7382 | 0.7377 | 0.7518 | 0.7246 | 0.476 | NA |
| H31x57 | consensus_cls_all | 0.3 | 0.7342 | 0.7334 | 0.7548 | 0.7135 | 0.4681 | NA |
| H31x57 | consensus_pvals_all | 0.1 | 0.8582 | 0.8584 | 0.8561 | 0.8603 | 0.7156 | 0.8873 |
| H31x57 | consensus_pvals_all | 0.15 | 0.8239 | 0.824 | 0.8213 | 0.8265 | 0.6475 | 0.8758 |
| H31x57 | consensus_pvals_all | 0.2 | 0.7933 | 0.7931 | 0.7977 | 0.7889 | 0.5862 | 0.8648 |
| H31x57 | consensus_pvals_all | 0.25 | 0.765 | 0.7648 | 0.7716 | 0.7584 | 0.5297 | 0.8541 |
| H31x57 | consensus_pvals_all | 0.3 | 0.7381 | 0.7379 | 0.7434 | 0.7327 | 0.4758 | 0.8440 |
| H31x57 | core-substituent_fps | 0.1 | 0.7803 | 0.7806 | 0.7737 | 0.7869 | 0.5602 | 0.8128 |
| H31x57 | core-substituent_fps | 0.15 | 0.7552 | 0.756 | 0.7431 | 0.7674 | 0.5103 | 0.8024 |
| H31x57 | core-substituent_fps | 0.2 | 0.7234 | 0.7236 | 0.7203 | 0.7266 | 0.4465 | 0.7928 |
| H31x57 | core-substituent_fps | 0.25 | 0.7004 | 0.7004 | 0.7006 | 0.7002 | 0.4004 | 0.7832 |
| H31x57 | core-substituent_fps | 0.3 | 0.6809 | 0.6808 | 0.683 | 0.6788 | 0.3614 | 0.7743 |
| H31x57 | deepsmiles_4_hashed_1024 | 0.1 | 0.7995 | 0.7997 | 0.7961 | 0.8029 | 0.5985 | 0.8377 |
| H31x57 | deepsmiles_4_hashed_1024 | 0.15 | 0.7668 | 0.7669 | 0.7645 | 0.7691 | 0.5332 | 0.8260 |
| H31x57 | deepsmiles_4_hashed_1024 | 0.2 | 0.7394 | 0.7394 | 0.7392 | 0.7397 | 0.4784 | 0.8152 |
| H31x57 | deepsmiles_4_hashed_1024 | 0.25 | 0.7177 | 0.7176 | 0.721 | 0.7145 | 0.4352 | 0.8054 |
| H31x57 | deepsmiles_4_hashed_1024 | 0.3 | 0.6967 | 0.6966 | 0.6987 | 0.6947 | 0.3932 | 0.7966 |
| H31x57 | morgan2_hashed_1024 | 0.1 | 0.8176 | 0.818 | 0.8114 | 0.8237 | 0.6345 | 0.8591 |
| H31x57 | morgan2_hashed_1024 | 0.15 | 0.7839 | 0.7843 | 0.7758 | 0.792 | 0.5677 | 0.8476 |
| H31x57 | morgan2_hashed_1024 | 0.2 | 0.7601 | 0.7604 | 0.7526 | 0.7676 | 0.5201 | 0.8372 |
| H31x57 | morgan2_hashed_1024 | 0.25 | 0.736 | 0.7363 | 0.7296 | 0.7424 | 0.4718 | 0.8278 |
| H31x57 | morgan2_hashed_1024 | 0.3 | 0.7295 | 0.7297 | 0.7261 | 0.733 | 0.4588 | 0.8212 |
| H31x57 | morgan4_hashed_1024 | 0.1 | 0.8025 | 0.8035 | 0.7897 | 0.8153 | 0.6048 | 0.8418 |
| H31x57 | morgan4_hashed_1024 | 0.15 | 0.773 | 0.7738 | 0.7615 | 0.7845 | 0.5457 | 0.8309 |
| H31x57 | morgan4_hashed_1024 | 0.2 | 0.7501 | 0.7505 | 0.7428 | 0.7573 | 0.4998 | 0.8210 |
| H31x57 | morgan4_hashed_1024 | 0.25 | 0.7287 | 0.729 | 0.7234 | 0.734 | 0.4572 | 0.8122 |
| H31x57 | morgan4_hashed_1024 | 0.3 | 0.7139 | 0.714 | 0.7103 | 0.7175 | 0.4275 | 0.8050 |
| H31x57 | ngram_4_hashed_1024 | 0.1 | 0.7894 | 0.7903 | 0.7781 | 0.8008 | 0.5786 | 0.8262 |
| H31x57 | ngram_4_hashed_1024 | 0.15 | 0.7645 | 0.765 | 0.7555 | 0.7736 | 0.5289 | 0.8154 |
| H31x57 | ngram_4_hashed_1024 | 0.2 | 0.7388 | 0.7389 | 0.7355 | 0.7421 | 0.4773 | 0.8058 |
| H31x57 | ngram_4_hashed_1024 | 0.25 | 0.7157 | 0.7159 | 0.7113 | 0.7202 | 0.4313 | 0.7972 |
| H31x57 | ngram_4_hashed_1024 | 0.3 | 0.6975 | 0.6977 | 0.6916 | 0.7034 | 0.3949 | 0.7889 |
| H31x57 | ngram_4_hashed_256 | 0.1 | 0.7925 | 0.7928 | 0.787 | 0.798 | 0.5846 | 0.8224 |
| H31x57 | ngram_4_hashed_256 | 0.15 | 0.76 | 0.76 | 0.761 | 0.7591 | 0.5195 | 0.8117 |
| H31x57 | ngram_4_hashed_256 | 0.2 | 0.7357 | 0.7356 | 0.7374 | 0.734 | 0.471 | 0.8025 |
| H31x57 | ngram_4_hashed_256 | 0.25 | 0.7176 | 0.7176 | 0.7191 | 0.7162 | 0.4349 | 0.7938 |
| H31x57 | ngram_4_hashed_256 | 0.3 | 0.6972 | 0.6971 | 0.6989 | 0.6954 | 0.394 | 0.7859 |
| H31x57 | ngram_4_hashed_64 | 0.1 | 0.7751 | 0.7753 | 0.771 | 0.7791 | 0.5497 | 0.8082 |
| H31x57 | ngram_4_hashed_64 | 0.15 | 0.7477 | 0.7476 | 0.7498 | 0.7455 | 0.4949 | 0.7979 |
| H31x57 | ngram_4_hashed_64 | 0.2 | 0.7286 | 0.7285 | 0.7301 | 0.7271 | 0.4568 | 0.7887 |
| H31x57 | ngram_4_hashed_64 | 0.25 | 0.7084 | 0.7085 | 0.7062 | 0.7106 | 0.4166 | 0.7804 |
| H31x57 | ngram_4_hashed_64 | 0.3 | 0.6881 | 0.6881 | 0.689 | 0.6872 | 0.376 | 0.7724 |
| H31x57 | ngram_6_hashed_1024 | 0.1 | 0.7945 | 0.7953 | 0.7843 | 0.8047 | 0.5884 | 0.8245 |
| H31x57 | ngram_6_hashed_1024 | 0.15 | 0.7645 | 0.7647 | 0.7619 | 0.7672 | 0.5285 | 0.8151 |
| H31x57 | ngram_6_hashed_1024 | 0.2 | 0.738 | 0.7381 | 0.7371 | 0.7389 | 0.4755 | 0.8060 |
| H31x57 | ngram_6_hashed_1024 | 0.25 | 0.7159 | 0.7159 | 0.7149 | 0.7169 | 0.4315 | 0.7973 |
| H31x57 | ngram_6_hashed_1024 | 0.3 | 0.6966 | 0.6966 | 0.6968 | 0.6963 | 0.3929 | 0.7891 |
| H31x57 | ngram_6_hashed_256 | 0.1 | 0.7919 | 0.7916 | 0.795 | 0.7887 | 0.5826 | 0.8201 |
| H31x57 | ngram_6_hashed_256 | 0.15 | 0.7594 | 0.7596 | 0.7568 | 0.7621 | 0.5185 | 0.8101 |
| H31x57 | ngram_6_hashed_256 | 0.2 | 0.7379 | 0.7378 | 0.7403 | 0.7356 | 0.4755 | 0.8012 |
| H31x57 | ngram_6_hashed_256 | 0.25 | 0.7132 | 0.7132 | 0.7124 | 0.714 | 0.4261 | 0.7925 |
| H31x57 | ngram_6_hashed_256 | 0.3 | 0.6953 | 0.6954 | 0.6946 | 0.6961 | 0.3904 | 0.7844 |
| H31x57 | ngram_6_hashed_64 | 0.1 | 0.7861 | 0.7859 | 0.7895 | 0.7827 | 0.5716 | 0.8197 |
| H31x57 | ngram_6_hashed_64 | 0.15 | 0.7588 | 0.7585 | 0.7664 | 0.7513 | 0.5172 | 0.8089 |
| H31x57 | ngram_6_hashed_64 | 0.2 | 0.732 | 0.7318 | 0.7382 | 0.7259 | 0.4638 | 0.7989 |
| H31x57 | ngram_6_hashed_64 | 0.25 | 0.7106 | 0.7104 | 0.7172 | 0.7041 | 0.421 | 0.7899 |
| H31x57 | ngram_6_hashed_64 | 0.3 | 0.6929 | 0.6927 | 0.6976 | 0.6882 | 0.3855 | 0.7814 |
| H31x57 | ngramsPE_4_hashed_1024 | 0.1 | 0.7934 | 0.7939 | 0.7869 | 0.7999 | 0.5859 | 0.8321 |
| H31x57 | ngramsPE_4_hashed_1024 | 0.15 | 0.7635 | 0.764 | 0.7545 | 0.7725 | 0.5267 | 0.8207 |
| H31x57 | ngramsPE_4_hashed_1024 | 0.2 | 0.7387 | 0.7389 | 0.7334 | 0.7439 | 0.477 | 0.8100 |
| H31x57 | ngramsPE_4_hashed_1024 | 0.25 | 0.7185 | 0.7186 | 0.7175 | 0.7196 | 0.4367 | 0.8007 |
| H31x57 | ngramsPE_4_hashed_1024 | 0.3 | 0.6996 | 0.6996 | 0.7002 | 0.699 | 0.3989 | 0.7925 |
| H31x57 | SMILES_extnd_connect_fps_2_1024 | 0.1 | 0.7973 | 0.7973 | 0.7965 | 0.7981 | 0.5944 | 0.8335 |
| H31x57 | SMILES_extnd_connect_fps_2_1024 | 0.15 | 0.7687 | 0.7685 | 0.7718 | 0.7655 | 0.537 | 0.8229 |
| H31x57 | SMILES_extnd_connect_fps_2_1024 | 0.2 | 0.7432 | 0.7431 | 0.7454 | 0.741 | 0.4861 | 0.8133 |
| H31x57 | SMILES_extnd_connect_fps_2_1024 | 0.25 | 0.7175 | 0.7174 | 0.7179 | 0.7171 | 0.4347 | 0.8043 |
| H31x57 | SMILES_extnd_connect_fps_2_1024 | 0.3 | 0.6995 | 0.6994 | 0.7002 | 0.6987 | 0.3987 | 0.7962 |
| H31x57 | SMILES_extnd_connect_fps_4_1024 | 0.1 | 0.8054 | 0.8055 | 0.802 | 0.8088 | 0.6107 | 0.8505 |
| H31x57 | SMILES_extnd_connect_fps_4_1024 | 0.15 | 0.7791 | 0.779 | 0.7829 | 0.7754 | 0.5579 | 0.8381 |
| H31x57 | SMILES_extnd_connect_fps_4_1024 | 0.2 | 0.7541 | 0.754 | 0.7578 | 0.7504 | 0.5079 | 0.8276 |
| H31x57 | SMILES_extnd_connect_fps_4_1024 | 0.25 | 0.729 | 0.729 | 0.7284 | 0.7295 | 0.4576 | 0.8179 |
| H31x57 | SMILES_extnd_connect_fps_4_1024 | 0.3 | 0.7098 | 0.7098 | 0.7104 | 0.7092 | 0.4193 | 0.8093 |
| H31x57 | selfies_4_hashed_1024 | 0.1 | 0.7905 | 0.7909 | 0.784 | 0.797 | 0.5806 | 0.8229 |
| H31x57 | selfies_4_hashed_1024 | 0.15 | 0.7622 | 0.7625 | 0.7554 | 0.7689 | 0.524 | 0.8126 |
| H31x57 | selfies_4_hashed_1024 | 0.2 | 0.7386 | 0.7386 | 0.738 | 0.7392 | 0.4769 | 0.8035 |
| H31x57 | selfies_4_hashed_1024 | 0.25 | 0.7148 | 0.7147 | 0.7174 | 0.7121 | 0.4292 | 0.7949 |
| H31x57 | selfies_4_hashed_1024 | 0.3 | 0.6951 | 0.695 | 0.6978 | 0.6924 | 0.39 | 0.7868 |
| H33x0123 | consensus_cls_all | 0.1 | 0.8808 | 0.846 | 0.9206 | 0.8411 | 0.4523 | NA |
| H33x0123 | consensus_cls_all | 0.15 | 0.822 | 0.7998 | 0.8467 | 0.7972 | 0.3359 | NA |
| H33x0123 | consensus_cls_all | 0.2 | 0.782 | 0.7584 | 0.8079 | 0.756 | 0.2675 | NA |
| H33x0123 | consensus_cls_all | 0.25 | 0.7692 | 0.7398 | 0.8015 | 0.7369 | 0.2468 | NA |
| H33x0123 | consensus_cls_all | 0.3 | 0.7711 | 0.7411 | 0.8041 | 0.7381 | 0.2475 | NA |
| H33x0123 | consensus_pvals_all | 0.1 | 0.9027 | 0.8659 | 0.9448 | 0.8607 | 0.4957 | 0.9327 |
| H33x0123 | consensus_pvals_all | 0.15 | 0.869 | 0.8401 | 0.9009 | 0.837 | 0.397 | 0.9254 |
| H33x0123 | consensus_pvals_all | 0.2 | 0.8189 | 0.8089 | 0.8299 | 0.8079 | 0.3251 | 0.9121 |
| H33x0123 | consensus_pvals_all | 0.25 | 0.7927 | 0.7835 | 0.8029 | 0.7825 | 0.2841 | 0.8982 |
| H33x0123 | consensus_pvals_all | 0.3 | 0.7787 | 0.7646 | 0.7941 | 0.7633 | 0.2603 | 0.8862 |
| H33x0123 | core-substituent_fps | 0.1 | 0.733 | 0.7387 | 0.7267 | 0.7393 | 0.2229 | 0.7700 |
| H33x0123 | core-substituent_fps | 0.15 | 0.7171 | 0.7243 | 0.7091 | 0.725 | 0.1954 | 0.7638 |
| H33x0123 | core-substituent_fps | 0.2 | 0.7073 | 0.7117 | 0.7025 | 0.7121 | 0.1856 | 0.7554 |
| H33x0123 | core-substituent_fps | 0.25 | 0.6863 | 0.6956 | 0.676 | 0.6965 | 0.1633 | 0.7482 |
| H33x0123 | core-substituent_fps | 0.3 | 0.6769 | 0.6854 | 0.6676 | 0.6862 | 0.1545 | 0.7415 |
| H33x0123 | deepsmiles_4_hashed_1024 | 0.1 | 0.7947 | 0.7753 | 0.8164 | 0.7729 | 0.304 | 0.8321 |
| H33x0123 | deepsmiles_4_hashed_1024 | 0.15 | 0.7554 | 0.7451 | 0.7668 | 0.744 | 0.2437 | 0.8218 |
| H33x0123 | deepsmiles_4_hashed_1024 | 0.2 | 0.731 | 0.7221 | 0.7407 | 0.7212 | 0.2126 | 0.8103 |
| H33x0123 | deepsmiles_4_hashed_1024 | 0.25 | 0.7036 | 0.7007 | 0.7067 | 0.7004 | 0.1811 | 0.7992 |
| H33x0123 | deepsmiles_4_hashed_1024 | 0.3 | 0.6959 | 0.6912 | 0.7011 | 0.6907 | 0.173 | 0.7896 |
| H33x0123 | morgan2_hashed_1024 | 0.1 | 0.7718 | 0.7628 | 0.7821 | 0.7616 | 0.29 | 0.8108 |
| H33x0123 | morgan2_hashed_1024 | 0.15 | 0.7625 | 0.7527 | 0.7735 | 0.7515 | 0.2629 | 0.8019 |
| H33x0123 | morgan2_hashed_1024 | 0.2 | 0.7378 | 0.7274 | 0.7492 | 0.7263 | 0.2228 | 0.7956 |
| H33x0123 | morgan2_hashed_1024 | 0.25 | 0.7196 | 0.7117 | 0.7283 | 0.7109 | 0.2001 | 0.7894 |
| H33x0123 | morgan2_hashed_1024 | 0.3 | 0.7087 | 0.7025 | 0.7154 | 0.7019 | 0.1854 | 0.7841 |
| H33x0123 | morgan4_hashed_1024 | 0.1 | 0.7589 | 0.7398 | 0.7806 | 0.7373 | 0.2667 | 0.8071 |
| H33x0123 | morgan4_hashed_1024 | 0.15 | 0.7392 | 0.7216 | 0.7589 | 0.7195 | 0.2336 | 0.7950 |
| H33x0123 | morgan4_hashed_1024 | 0.2 | 0.7248 | 0.6969 | 0.7559 | 0.6937 | 0.2106 | 0.7853 |
| H33x0123 | morgan4_hashed_1024 | 0.25 | 0.7084 | 0.687 | 0.7319 | 0.6848 | 0.1877 | 0.7776 |
| H33x0123 | morgan4_hashed_1024 | 0.3 | 0.6841 | 0.6793 | 0.6894 | 0.6788 | 0.1622 | 0.7704 |
| H33x0123 | ngram_4_hashed_1024 | 0.1 | 0.7889 | 0.7576 | 0.8246 | 0.7533 | 0.304 | 0.8265 |
| H33x0123 | ngram_4_hashed_1024 | 0.15 | 0.7604 | 0.7485 | 0.7739 | 0.747 | 0.2592 | 0.8169 |
| H33x0123 | ngram_4_hashed_1024 | 0.2 | 0.739 | 0.744 | 0.7335 | 0.7445 | 0.2279 | 0.8066 |
| H33x0123 | ngram_4_hashed_1024 | 0.25 | 0.7167 | 0.7296 | 0.7026 | 0.7309 | 0.2011 | 0.7970 |
| H33x0123 | ngram_4_hashed_1024 | 0.3 | 0.7154 | 0.7272 | 0.7023 | 0.7284 | 0.1973 | 0.7909 |
| H33x0123 | ngram_4_hashed_256 | 0.1 | 0.7935 | 0.7695 | 0.8206 | 0.7663 | 0.3095 | 0.8352 |
| H33x0123 | ngram_4_hashed_256 | 0.15 | 0.7547 | 0.7465 | 0.7638 | 0.7455 | 0.2496 | 0.8231 |
| H33x0123 | ngram_4_hashed_256 | 0.2 | 0.7175 | 0.7212 | 0.7134 | 0.7216 | 0.2029 | 0.8114 |
| H33x0123 | ngram_4_hashed_256 | 0.25 | 0.7006 | 0.7062 | 0.6944 | 0.7067 | 0.1829 | 0.7998 |
| H33x0123 | ngram_4_hashed_256 | 0.3 | 0.682 | 0.6929 | 0.6701 | 0.6939 | 0.1621 | 0.7902 |
| H33x0123 | ngram_4_hashed_64 | 0.1 | 0.7854 | 0.7591 | 0.8148 | 0.7559 | 0.2917 | 0.8269 |
| H33x0123 | ngram_4_hashed_64 | 0.15 | 0.7607 | 0.7385 | 0.7854 | 0.736 | 0.2499 | 0.8181 |
| H33x0123 | ngram_4_hashed_64 | 0.2 | 0.7385 | 0.7177 | 0.7614 | 0.7155 | 0.2198 | 0.8091 |
| H33x0123 | ngram_4_hashed_64 | 0.25 | 0.7211 | 0.7054 | 0.7384 | 0.7039 | 0.1964 | 0.8005 |
| H33x0123 | ngram_4_hashed_64 | 0.3 | 0.7024 | 0.6934 | 0.7124 | 0.6925 | 0.1783 | 0.7929 |
| H33x0123 | ngram_6_hashed_1024 | 0.1 | 0.794 | 0.7764 | 0.8139 | 0.7741 | 0.3116 | 0.8400 |
| H33x0123 | ngram_6_hashed_1024 | 0.15 | 0.7672 | 0.757 | 0.7786 | 0.7558 | 0.2657 | 0.8284 |
| H33x0123 | ngram_6_hashed_1024 | 0.2 | 0.7427 | 0.7355 | 0.7508 | 0.7347 | 0.2303 | 0.8185 |
| H33x0123 | ngram_6_hashed_1024 | 0.25 | 0.7139 | 0.7227 | 0.7041 | 0.7236 | 0.1962 | 0.8085 |
| H33x0123 | ngram_6_hashed_1024 | 0.3 | 0.7099 | 0.7219 | 0.6966 | 0.7231 | 0.1918 | 0.8015 |
| H33x0123 | ngram_6_hashed_256 | 0.1 | 0.81 | 0.7866 | 0.8364 | 0.7837 | 0.3232 | 0.8552 |
| H33x0123 | ngram_6_hashed_256 | 0.15 | 0.7788 | 0.7623 | 0.797 | 0.7605 | 0.27 | 0.8436 |
| H33x0123 | ngram_6_hashed_256 | 0.2 | 0.7491 | 0.7357 | 0.7638 | 0.7344 | 0.2293 | 0.8334 |
| H33x0123 | ngram_6_hashed_256 | 0.25 | 0.7166 | 0.7184 | 0.7147 | 0.7185 | 0.1954 | 0.8229 |
| H33x0123 | ngram_6_hashed_256 | 0.3 | 0.7061 | 0.7052 | 0.7071 | 0.7051 | 0.1829 | 0.8142 |
| H33x0123 | ngram_6_hashed_64 | 0.1 | 0.7789 | 0.7525 | 0.8088 | 0.749 | 0.291 | 0.8308 |
| H33x0123 | ngram_6_hashed_64 | 0.15 | 0.7547 | 0.7359 | 0.7755 | 0.7338 | 0.2442 | 0.8191 |
| H33x0123 | ngram_6_hashed_64 | 0.2 | 0.7294 | 0.7281 | 0.7309 | 0.7279 | 0.2143 | 0.8061 |
| H33x0123 | ngram_6_hashed_64 | 0.25 | 0.7094 | 0.7092 | 0.7097 | 0.7092 | 0.1897 | 0.7951 |
| H33x0123 | ngram_6_hashed_64 | 0.3 | 0.6877 | 0.6915 | 0.6834 | 0.6919 | 0.1665 | 0.7854 |
| H33x0123 | ngramsPE_4_hashed_1024 | 0.1 | 0.7752 | 0.7723 | 0.7783 | 0.772 | 0.2822 | 0.8320 |
| H33x0123 | ngramsPE_4_hashed_1024 | 0.15 | 0.7462 | 0.7545 | 0.7371 | 0.7554 | 0.2406 | 0.8165 |
| H33x0123 | ngramsPE_4_hashed_1024 | 0.2 | 0.7214 | 0.7302 | 0.7117 | 0.7311 | 0.2017 | 0.8041 |
| H33x0123 | ngramsPE_4_hashed_1024 | 0.25 | 0.7091 | 0.7028 | 0.716 | 0.7022 | 0.1847 | 0.7928 |
| H33x0123 | ngramsPE_4_hashed_1024 | 0.3 | 0.6873 | 0.6871 | 0.6875 | 0.687 | 0.1636 | 0.7827 |
| H33x0123 | SMILES_extnd_connect_fps_2_1024 | 0.1 | 0.8292 | 0.8185 | 0.8411 | 0.8172 | 0.3563 | 0.8649 |
| H33x0123 | SMILES_extnd_connect_fps_2_1024 | 0.15 | 0.8035 | 0.793 | 0.8152 | 0.7919 | 0.304 | 0.8569 |
| H33x0123 | SMILES_extnd_connect_fps_2_1024 | 0.2 | 0.7747 | 0.7692 | 0.7807 | 0.7686 | 0.2603 | 0.8493 |
| H33x0123 | SMILES_extnd_connect_fps_2_1024 | 0.25 | 0.7443 | 0.7457 | 0.7428 | 0.7458 | 0.2269 | 0.8421 |
| H33x0123 | SMILES_extnd_connect_fps_2_1024 | 0.3 | 0.7549 | 0.7606 | 0.7486 | 0.7612 | 0.2412 | 0.8388 |
| H33x0123 | SMILES_extnd_connect_fps_4_1024 | 0.1 | 0.8283 | 0.8331 | 0.8228 | 0.8337 | 0.355 | 0.8699 |
| H33x0123 | SMILES_extnd_connect_fps_4_1024 | 0.15 | 0.7987 | 0.7988 | 0.7987 | 0.7988 | 0.3005 | 0.8599 |
| H33x0123 | SMILES_extnd_connect_fps_4_1024 | 0.2 | 0.7693 | 0.7679 | 0.7708 | 0.7678 | 0.2582 | 0.8506 |
| H33x0123 | SMILES_extnd_connect_fps_4_1024 | 0.25 | 0.748 | 0.7444 | 0.752 | 0.744 | 0.2308 | 0.8425 |
| H33x0123 | SMILES_extnd_connect_fps_4_1024 | 0.3 | 0.754 | 0.7527 | 0.7554 | 0.7526 | 0.2371 | 0.8386 |
| H33x0123 | selfies_4_hashed_1024 | 0.1 | 0.7739 | 0.7651 | 0.7837 | 0.7641 | 0.281 | 0.8082 |
| H33x0123 | selfies_4_hashed_1024 | 0.15 | 0.7416 | 0.745 | 0.7379 | 0.7454 | 0.2329 | 0.7988 |
| H33x0123 | selfies_4_hashed_1024 | 0.2 | 0.7194 | 0.7196 | 0.7193 | 0.7196 | 0.1997 | 0.7894 |
| H33x0123 | selfies_4_hashed_1024 | 0.25 | 0.6915 | 0.6982 | 0.684 | 0.6989 | 0.1698 | 0.7801 |
| H33x0123 | selfies_4_hashed_1024 | 0.3 | 0.6689 | 0.6831 | 0.6533 | 0.6845 | 0.1494 | 0.7707 |
| H33x45 | consensus_cls_all | 0.1 | 0.8652 | 0.8648 | 0.8691 | 0.8614 | 0.7284 | NA |
| H33x45 | consensus_cls_all | 0.15 | 0.8151 | 0.815 | 0.8158 | 0.8143 | 0.6292 | NA |
| H33x45 | consensus_cls_all | 0.2 | 0.7822 | 0.7811 | 0.7962 | 0.7681 | 0.5628 | NA |
| H33x45 | consensus_cls_all | 0.25 | 0.771 | 0.7698 | 0.7868 | 0.7553 | 0.5404 | NA |
| H33x45 | consensus_cls_all | 0.3 | 0.774 | 0.7724 | 0.7938 | 0.7541 | 0.5462 | NA |
| H33x45 | consensus_pvals_all | 0.1 | 0.8792 | 0.8794 | 0.8766 | 0.8817 | 0.7569 | 0.9081 |
| H33x45 | consensus_pvals_all | 0.15 | 0.8444 | 0.8443 | 0.8457 | 0.843 | 0.6873 | 0.8976 |
| H33x45 | consensus_pvals_all | 0.2 | 0.8098 | 0.8091 | 0.817 | 0.8025 | 0.6177 | 0.8877 |
| H33x45 | consensus_pvals_all | 0.25 | 0.7809 | 0.7805 | 0.7857 | 0.7761 | 0.5603 | 0.8784 |
| H33x45 | consensus_pvals_all | 0.3 | 0.7862 | 0.7856 | 0.7933 | 0.779 | 0.5707 | 0.8737 |
| H33x45 | core-substituent_fps | 0.1 | 0.8058 | 0.8053 | 0.8111 | 0.8004 | 0.6097 | 0.8473 |
| H33x45 | core-substituent_fps | 0.15 | 0.7779 | 0.777 | 0.7877 | 0.7681 | 0.5538 | 0.8339 |
| H33x45 | core-substituent_fps | 0.2 | 0.7565 | 0.7557 | 0.7652 | 0.7477 | 0.5111 | 0.8236 |
| H33x45 | core-substituent_fps | 0.25 | 0.732 | 0.731 | 0.7445 | 0.7196 | 0.4625 | 0.8147 |
| H33x45 | core-substituent_fps | 0.3 | 0.7282 | 0.7268 | 0.7448 | 0.7115 | 0.4549 | 0.8088 |
| H33x45 | deepsmiles_4_hashed_1024 | 0.1 | 0.8172 | 0.8181 | 0.8095 | 0.8248 | 0.632 | 0.8628 |
| H33x45 | deepsmiles_4_hashed_1024 | 0.15 | 0.7892 | 0.7893 | 0.7884 | 0.79 | 0.5765 | 0.8503 |
| H33x45 | deepsmiles_4_hashed_1024 | 0.2 | 0.7686 | 0.7685 | 0.7695 | 0.7677 | 0.5356 | 0.8406 |
| H33x45 | deepsmiles_4_hashed_1024 | 0.25 | 0.7397 | 0.7395 | 0.7425 | 0.7368 | 0.4782 | 0.8317 |
| H33x45 | deepsmiles_4_hashed_1024 | 0.3 | 0.7499 | 0.7499 | 0.7502 | 0.7496 | 0.4984 | 0.8275 |
| H33x45 | morgan2_hashed_1024 | 0.1 | 0.8409 | 0.8401 | 0.8523 | 0.8294 | 0.6803 | 0.8779 |
| H33x45 | morgan2_hashed_1024 | 0.15 | 0.8057 | 0.8053 | 0.8116 | 0.7999 | 0.6103 | 0.8682 |
| H33x45 | morgan2_hashed_1024 | 0.2 | 0.7785 | 0.7782 | 0.7821 | 0.7748 | 0.5556 | 0.8598 |
| H33x45 | morgan2_hashed_1024 | 0.25 | 0.7579 | 0.758 | 0.7566 | 0.7593 | 0.5148 | 0.8523 |
| H33x45 | morgan2_hashed_1024 | 0.3 | 0.7785 | 0.7784 | 0.7795 | 0.7775 | 0.5558 | 0.8505 |
| H33x45 | morgan4_hashed_1024 | 0.1 | 0.8283 | 0.8292 | 0.819 | 0.8376 | 0.6557 | 0.8650 |
| H33x45 | morgan4_hashed_1024 | 0.15 | 0.8023 | 0.8025 | 0.7989 | 0.8056 | 0.6035 | 0.8553 |
| H33x45 | morgan4_hashed_1024 | 0.2 | 0.7736 | 0.7738 | 0.7708 | 0.7764 | 0.5461 | 0.8473 |
| H33x45 | morgan4_hashed_1024 | 0.25 | 0.7506 | 0.7506 | 0.7507 | 0.7504 | 0.5001 | 0.8399 |
| H33x45 | morgan4_hashed_1024 | 0.3 | 0.767 | 0.767 | 0.7666 | 0.7674 | 0.533 | 0.8373 |
| H33x45 | ngram_4_hashed_1024 | 0.1 | 0.8233 | 0.825 | 0.8076 | 0.839 | 0.6462 | 0.8602 |
| H33x45 | ngram_4_hashed_1024 | 0.15 | 0.7939 | 0.7956 | 0.7772 | 0.8106 | 0.5874 | 0.8500 |
| H33x45 | ngram_4_hashed_1024 | 0.2 | 0.7645 | 0.7655 | 0.7532 | 0.7758 | 0.5283 | 0.8406 |
| H33x45 | ngram_4_hashed_1024 | 0.25 | 0.7426 | 0.7435 | 0.7305 | 0.7548 | 0.4848 | 0.8322 |
| H33x45 | ngram_4_hashed_1024 | 0.3 | 0.7546 | 0.7554 | 0.746 | 0.7633 | 0.5083 | 0.8287 |
| H33x45 | ngram_4_hashed_256 | 0.1 | 0.8127 | 0.8133 | 0.8087 | 0.8168 | 0.6226 | 0.8371 |
| H33x45 | ngram_4_hashed_256 | 0.15 | 0.7818 | 0.7825 | 0.7746 | 0.789 | 0.5623 | 0.8294 |
| H33x45 | ngram_4_hashed_256 | 0.2 | 0.7602 | 0.7605 | 0.7568 | 0.7636 | 0.5187 | 0.8217 |
| H33x45 | ngram_4_hashed_256 | 0.25 | 0.7447 | 0.7445 | 0.7474 | 0.742 | 0.4883 | 0.8149 |
| H33x45 | ngram_4_hashed_256 | 0.3 | 0.7521 | 0.7514 | 0.7612 | 0.743 | 0.5027 | 0.8116 |
| H33x45 | ngram_4_hashed_64 | 0.1 | 0.8173 | 0.8155 | 0.8333 | 0.8013 | 0.631 | 0.8454 |
| H33x45 | ngram_4_hashed_64 | 0.15 | 0.779 | 0.7789 | 0.7801 | 0.7779 | 0.5559 | 0.8350 |
| H33x45 | ngram_4_hashed_64 | 0.2 | 0.7514 | 0.7519 | 0.7461 | 0.7567 | 0.5018 | 0.8255 |
| H33x45 | ngram_4_hashed_64 | 0.25 | 0.7279 | 0.7278 | 0.7291 | 0.7267 | 0.4545 | 0.8165 |
| H33x45 | ngram_4_hashed_64 | 0.3 | 0.7335 | 0.7333 | 0.7361 | 0.7309 | 0.4657 | 0.8116 |
| H33x45 | ngram_6_hashed_1024 | 0.1 | 0.8149 | 0.8148 | 0.8154 | 0.8143 | 0.6271 | 0.8443 |
| H33x45 | ngram_6_hashed_1024 | 0.15 | 0.7872 | 0.7873 | 0.7868 | 0.7877 | 0.573 | 0.8354 |
| H33x45 | ngram_6_hashed_1024 | 0.2 | 0.7626 | 0.7626 | 0.7629 | 0.7624 | 0.5239 | 0.8270 |
| H33x45 | ngram_6_hashed_1024 | 0.25 | 0.7379 | 0.7375 | 0.7424 | 0.7334 | 0.4746 | 0.8193 |
| H33x45 | ngram_6_hashed_1024 | 0.3 | 0.7443 | 0.7438 | 0.7519 | 0.7368 | 0.4874 | 0.8153 |
| H33x45 | ngram_6_hashed_256 | 0.1 | 0.8117 | 0.8117 | 0.8119 | 0.8114 | 0.6211 | 0.8349 |
| H33x45 | ngram_6_hashed_256 | 0.15 | 0.7885 | 0.7884 | 0.7892 | 0.7877 | 0.5747 | 0.8283 |
| H33x45 | ngram_6_hashed_256 | 0.2 | 0.7611 | 0.7611 | 0.7607 | 0.7615 | 0.5207 | 0.8216 |
| H33x45 | ngram_6_hashed_256 | 0.25 | 0.7285 | 0.7284 | 0.7296 | 0.7273 | 0.4556 | 0.8145 |
| H33x45 | ngram_6_hashed_256 | 0.3 | 0.7242 | 0.7242 | 0.7238 | 0.7245 | 0.4471 | 0.8096 |
| H33x45 | ngram_6_hashed_64 | 0.1 | 0.8121 | 0.8121 | 0.8123 | 0.812 | 0.6218 | 0.8406 |
| H33x45 | ngram_6_hashed_64 | 0.15 | 0.7828 | 0.7834 | 0.7776 | 0.7881 | 0.564 | 0.8319 |
| H33x45 | ngram_6_hashed_64 | 0.2 | 0.7498 | 0.7502 | 0.7456 | 0.754 | 0.4983 | 0.8234 |
| H33x45 | ngram_6_hashed_64 | 0.25 | 0.7288 | 0.7293 | 0.7233 | 0.7344 | 0.4566 | 0.8152 |
| H33x45 | ngram_6_hashed_64 | 0.3 | 0.7209 | 0.7214 | 0.7142 | 0.7276 | 0.441 | 0.8087 |
| H33x45 | ngramsPE_4_hashed_1024 | 0.1 | 0.8205 | 0.8201 | 0.8256 | 0.8154 | 0.6394 | 0.8588 |
| H33x45 | ngramsPE_4_hashed_1024 | 0.15 | 0.787 | 0.7873 | 0.7835 | 0.7904 | 0.5726 | 0.8458 |
| H33x45 | ngramsPE_4_hashed_1024 | 0.2 | 0.7628 | 0.7635 | 0.7548 | 0.7708 | 0.5247 | 0.8360 |
| H33x45 | ngramsPE_4_hashed_1024 | 0.25 | 0.7382 | 0.7389 | 0.7288 | 0.7476 | 0.4758 | 0.8275 |
| H33x45 | ngramsPE_4_hashed_1024 | 0.3 | 0.7327 | 0.7333 | 0.7245 | 0.7409 | 0.4647 | 0.8218 |
| H33x45 | SMILES_extnd_connect_fps_2_1024 | 0.1 | 0.8178 | 0.8182 | 0.8079 | 0.8276 | 0.6356 | 0.8573 |
| H33x45 | SMILES_extnd_connect_fps_2_1024 | 0.15 | 0.7866 | 0.7872 | 0.7788 | 0.7945 | 0.5728 | 0.8465 |
| H33x45 | SMILES_extnd_connect_fps_2_1024 | 0.2 | 0.7603 | 0.7607 | 0.755 | 0.7655 | 0.5196 | 0.8375 |
| H33x45 | SMILES_extnd_connect_fps_2_1024 | 0.25 | 0.735 | 0.7355 | 0.7296 | 0.7405 | 0.4692 | 0.8294 |
| H33x45 | SMILES_extnd_connect_fps_2_1024 | 0.3 | 0.7452 | 0.7453 | 0.7426 | 0.7477 | 0.4894 | 0.8259 |
| H33x45 | SMILES_extnd_connect_fps_4_1024 | 0.1 | 0.8321 | 0.8324 | 0.8273 | 0.837 | 0.6639 | 0.8743 |
| H33x45 | SMILES_extnd_connect_fps_4_1024 | 0.15 | 0.798 | 0.7982 | 0.7957 | 0.8004 | 0.5954 | 0.8625 |
| H33x45 | SMILES_extnd_connect_fps_4_1024 | 0.2 | 0.7785 | 0.7784 | 0.7797 | 0.7772 | 0.5556 | 0.8529 |
| H33x45 | SMILES_extnd_connect_fps_4_1024 | 0.25 | 0.759 | 0.7588 | 0.7616 | 0.7564 | 0.5168 | 0.8454 |
| H33x45 | SMILES_extnd_connect_fps_4_1024 | 0.3 | 0.7743 | 0.7742 | 0.7751 | 0.7735 | 0.5474 | 0.8429 |
| H33x45 | selfies_4_hashed_1024 | 0.1 | 0.8115 | 0.8112 | 0.8143 | 0.8087 | 0.6201 | 0.8499 |
| H33x45 | selfies_4_hashed_1024 | 0.15 | 0.7783 | 0.7782 | 0.7788 | 0.7778 | 0.5549 | 0.8388 |
| H33x45 | selfies_4_hashed_1024 | 0.2 | 0.7548 | 0.7544 | 0.7588 | 0.7507 | 0.5081 | 0.8293 |
| H33x45 | selfies_4_hashed_1024 | 0.25 | 0.735 | 0.7346 | 0.7398 | 0.7302 | 0.4687 | 0.8205 |
| H33x45 | selfies_4_hashed_1024 | 0.3 | 0.7356 | 0.7354 | 0.7386 | 0.7327 | 0.4701 | 0.8155 |
| H34x | core-substituent_fps | 0.1 | 0.7484 | 0.7291 | 0.7697 | 0.7271 | 0.231 | 0.7869 |
| H34x | core-substituent_fps | 0.15 | 0.7335 | 0.7227 | 0.7454 | 0.7217 | 0.2086 | 0.7765 |
| H34x | core-substituent_fps | 0.2 | 0.7292 | 0.7102 | 0.75 | 0.7085 | 0.1995 | 0.7688 |
| H34x | core-substituent_fps | 0.25 | 0.7036 | 0.6926 | 0.7157 | 0.6916 | 0.1767 | 0.7622 |
| H34x | core-substituent_fps | 0.3 | 0.6831 | 0.6787 | 0.6878 | 0.6783 | 0.1588 | 0.7555 |
| H34x | deepsmiles_4_hashed_1024 | 0.1 | 0.7864 | 0.7706 | 0.804 | 0.7688 | 0.2875 | 0.8447 |
| H34x | deepsmiles_4_hashed_1024 | 0.15 | 0.7592 | 0.7481 | 0.7715 | 0.7469 | 0.2512 | 0.8265 |
| H34x | deepsmiles_4_hashed_1024 | 0.2 | 0.7368 | 0.7271 | 0.7476 | 0.7261 | 0.2205 | 0.8143 |
| H34x | deepsmiles_4_hashed_1024 | 0.25 | 0.7142 | 0.7028 | 0.7268 | 0.7016 | 0.1944 | 0.8037 |
| H34x | deepsmiles_4_hashed_1024 | 0.3 | 0.7006 | 0.691 | 0.7113 | 0.69 | 0.1782 | 0.7951 |
| H34x | morgan2_hashed_1024 | 0.1 | 0.8039 | 0.7898 | 0.8198 | 0.788 | 0.3223 | 0.8304 |
| H34x | morgan2_hashed_1024 | 0.15 | 0.7604 | 0.7568 | 0.7645 | 0.7564 | 0.2586 | 0.8209 |
| H34x | morgan2_hashed_1024 | 0.2 | 0.7367 | 0.7303 | 0.7438 | 0.7296 | 0.2235 | 0.8112 |
| H34x | morgan2_hashed_1024 | 0.25 | 0.717 | 0.7149 | 0.7194 | 0.7146 | 0.1978 | 0.8023 |
| H34x | morgan2_hashed_1024 | 0.3 | 0.7037 | 0.7048 | 0.7026 | 0.7049 | 0.1821 | 0.7951 |
| H34x | morgan4_hashed_1024 | 0.1 | 0.7573 | 0.7454 | 0.7707 | 0.7439 | 0.2645 | 0.8107 |
| H34x | morgan4_hashed_1024 | 0.15 | 0.7312 | 0.7224 | 0.7409 | 0.7214 | 0.2212 | 0.7976 |
| H34x | morgan4_hashed_1024 | 0.2 | 0.7116 | 0.7091 | 0.7143 | 0.7089 | 0.194 | 0.7862 |
| H34x | morgan4_hashed_1024 | 0.25 | 0.6814 | 0.6984 | 0.6627 | 0.7002 | 0.1635 | 0.7748 |
| H34x | morgan4_hashed_1024 | 0.3 | 0.6586 | 0.6858 | 0.6286 | 0.6886 | 0.1411 | 0.7644 |
| H34x | ngram_4_hashed_1024 | 0.1 | 0.8185 | 0.7786 | 0.8634 | 0.7736 | 0.3343 | 0.8566 |
| H34x | ngram_4_hashed_1024 | 0.15 | 0.7823 | 0.7589 | 0.8085 | 0.7562 | 0.2796 | 0.8457 |
| H34x | ngram_4_hashed_1024 | 0.2 | 0.7445 | 0.7415 | 0.7477 | 0.7412 | 0.2336 | 0.8345 |
| H34x | ngram_4_hashed_1024 | 0.25 | 0.7173 | 0.7246 | 0.7092 | 0.7253 | 0.2006 | 0.8238 |
| H34x | ngram_4_hashed_1024 | 0.3 | 0.7138 | 0.7238 | 0.7029 | 0.7248 | 0.195 | 0.8161 |
| H34x | ngram_4_hashed_256 | 0.1 | 0.7964 | 0.7641 | 0.8333 | 0.7594 | 0.3235 | 0.8371 |
| H34x | ngram_4_hashed_256 | 0.15 | 0.7678 | 0.7393 | 0.8 | 0.7357 | 0.2708 | 0.8276 |
| H34x | ngram_4_hashed_256 | 0.2 | 0.7489 | 0.7154 | 0.7859 | 0.7118 | 0.2331 | 0.8203 |
| H34x | ngram_4_hashed_256 | 0.25 | 0.7263 | 0.6985 | 0.757 | 0.6955 | 0.2062 | 0.8120 |
| H34x | ngram_4_hashed_256 | 0.3 | 0.7155 | 0.6927 | 0.7406 | 0.6904 | 0.1918 | 0.8055 |
| H34x | ngram_4_hashed_64 | 0.1 | 0.778 | 0.7429 | 0.8178 | 0.7382 | 0.289 | 0.8166 |
| H34x | ngram_4_hashed_64 | 0.15 | 0.7547 | 0.7341 | 0.7778 | 0.7316 | 0.2553 | 0.8053 |
| H34x | ngram_4_hashed_64 | 0.2 | 0.7262 | 0.7203 | 0.7327 | 0.7196 | 0.2137 | 0.7967 |
| H34x | ngram_4_hashed_64 | 0.25 | 0.7072 | 0.7067 | 0.7079 | 0.7066 | 0.1893 | 0.7884 |
| H34x | ngram_4_hashed_64 | 0.3 | 0.7015 | 0.7026 | 0.7003 | 0.7027 | 0.1802 | 0.7816 |
| H34x | ngram_6_hashed_1024 | 0.1 | 0.8067 | 0.773 | 0.8447 | 0.7687 | 0.321 | 0.8492 |
| H34x | ngram_6_hashed_1024 | 0.15 | 0.7767 | 0.7484 | 0.8083 | 0.7452 | 0.2695 | 0.8404 |
| H34x | ngram_6_hashed_1024 | 0.2 | 0.738 | 0.7279 | 0.7492 | 0.7268 | 0.2227 | 0.8295 |
| H34x | ngram_6_hashed_1024 | 0.25 | 0.7177 | 0.7074 | 0.7291 | 0.7063 | 0.1954 | 0.8195 |
| H34x | ngram_6_hashed_1024 | 0.3 | 0.7 | 0.6979 | 0.7023 | 0.6977 | 0.1776 | 0.8100 |
| H34x | ngram_6_hashed_256 | 0.1 | 0.8006 | 0.7798 | 0.8241 | 0.7772 | 0.3132 | 0.8301 |
| H34x | ngram_6_hashed_256 | 0.15 | 0.7769 | 0.7582 | 0.7976 | 0.7563 | 0.2658 | 0.8247 |
| H34x | ngram_6_hashed_256 | 0.2 | 0.7466 | 0.7342 | 0.7601 | 0.733 | 0.2264 | 0.8176 |
| H34x | ngram_6_hashed_256 | 0.25 | 0.7297 | 0.7226 | 0.7376 | 0.7219 | 0.2065 | 0.8093 |
| H34x | ngram_6_hashed_256 | 0.3 | 0.7138 | 0.7084 | 0.7197 | 0.7079 | 0.1895 | 0.8022 |
| H34x | ngram_6_hashed_64 | 0.1 | 0.7401 | 0.7548 | 0.7236 | 0.7565 | 0.2401 | 0.7776 |
| H34x | ngram_6_hashed_64 | 0.15 | 0.7221 | 0.7307 | 0.7126 | 0.7316 | 0.2093 | 0.7690 |
| H34x | ngram_6_hashed_64 | 0.2 | 0.7033 | 0.7186 | 0.6865 | 0.7202 | 0.1877 | 0.7596 |
| H34x | ngram_6_hashed_64 | 0.25 | 0.6794 | 0.7004 | 0.6562 | 0.7026 | 0.1619 | 0.7511 |
| H34x | ngram_6_hashed_64 | 0.3 | 0.6645 | 0.6833 | 0.6438 | 0.6851 | 0.145 | 0.7440 |
| H34x | ngramsPE_4_hashed_1024 | 0.1 | 0.8077 | 0.7727 | 0.8466 | 0.7689 | 0.3024 | 0.8583 |
| H34x | ngramsPE_4_hashed_1024 | 0.15 | 0.7541 | 0.7489 | 0.7598 | 0.7483 | 0.2432 | 0.8398 |
| H34x | ngramsPE_4_hashed_1024 | 0.2 | 0.7166 | 0.7241 | 0.7082 | 0.7249 | 0.2001 | 0.8235 |
| H34x | ngramsPE_4_hashed_1024 | 0.25 | 0.6997 | 0.7086 | 0.6901 | 0.7094 | 0.1788 | 0.8097 |
| H34x | ngramsPE_4_hashed_1024 | 0.3 | 0.6799 | 0.6935 | 0.6649 | 0.6948 | 0.1591 | 0.7984 |
| H34x | SMILES_extnd_connect_fps_2_1024 | 0.1 | 0.8384 | 0.8196 | 0.8594 | 0.8174 | 0.3628 | 0.8834 |
| H34x | SMILES_extnd_connect_fps_2_1024 | 0.15 | 0.8024 | 0.7928 | 0.8129 | 0.7918 | 0.3037 | 0.8736 |
| H34x | SMILES_extnd_connect_fps_2_1024 | 0.2 | 0.7805 | 0.7753 | 0.7861 | 0.7748 | 0.2693 | 0.8648 |
| H34x | SMILES_extnd_connect_fps_2_1024 | 0.25 | 0.7528 | 0.7518 | 0.754 | 0.7517 | 0.2348 | 0.8567 |
| H34x | SMILES_extnd_connect_fps_2_1024 | 0.3 | 0.7645 | 0.7689 | 0.7597 | 0.7693 | 0.2521 | 0.8534 |
| H34x | SMILES_extnd_connect_fps_4_1024 | 0.1 | 0.8208 | 0.8153 | 0.827 | 0.8147 | 0.3348 | 0.8609 |
| H34x | SMILES_extnd_connect_fps_4_1024 | 0.15 | 0.7943 | 0.7841 | 0.8056 | 0.7831 | 0.2874 | 0.8519 |
| H34x | SMILES_extnd_connect_fps_4_1024 | 0.2 | 0.7722 | 0.769 | 0.7758 | 0.7687 | 0.2577 | 0.8424 |
| H34x | SMILES_extnd_connect_fps_4_1024 | 0.25 | 0.7478 | 0.7501 | 0.7453 | 0.7503 | 0.2286 | 0.8345 |
| H34x | SMILES_extnd_connect_fps_4_1024 | 0.3 | 0.7602 | 0.7629 | 0.7571 | 0.7632 | 0.2418 | 0.8312 |
| H34x | selfies_4_hashed_1024 | 0.1 | 0.7883 | 0.789 | 0.7874 | 0.7891 | 0.2954 | 0.8332 |
| H34x | selfies_4_hashed_1024 | 0.15 | 0.7505 | 0.7518 | 0.749 | 0.752 | 0.2376 | 0.8204 |
| H34x | selfies_4_hashed_1024 | 0.2 | 0.7254 | 0.7233 | 0.7276 | 0.7231 | 0.2044 | 0.8091 |
| H34x | selfies_4_hashed_1024 | 0.25 | 0.7048 | 0.6989 | 0.7112 | 0.6983 | 0.1807 | 0.7985 |
| H34x | selfies_4_hashed_1024 | 0.3 | 0.6859 | 0.6805 | 0.6919 | 0.68 | 0.1621 | 0.7886 |
| H34x | consensus_cls_all | 0.1 | 0.8881 | 0.8426 | 0.9394 | 0.8367 | 0.4452 | NA |
| H34x | consensus_cls_all | 0.15 | 0.824 | 0.8001 | 0.8505 | 0.7974 | 0.3367 | NA |
| H34x | consensus_cls_all | 0.2 | 0.7778 | 0.7618 | 0.7955 | 0.7602 | 0.2661 | NA |
| H34x | consensus_cls_all | 0.25 | 0.7561 | 0.7382 | 0.7758 | 0.7364 | 0.2335 | NA |
| H34x | consensus_cls_all | 0.3 | 0.7532 | 0.7392 | 0.7684 | 0.7379 | 0.2315 | NA |
| H34x | consensus_pvals_all | 0.1 | 0.8978 | 0.8622 | 0.9382 | 0.8573 | 0.4837 | 0.9375 |
| H34x | consensus_pvals_all | 0.15 | 0.8691 | 0.8386 | 0.9031 | 0.8352 | 0.4007 | 0.9274 |
| H34x | consensus_pvals_all | 0.2 | 0.8288 | 0.8109 | 0.8486 | 0.809 | 0.3332 | 0.9142 |
| H34x | consensus_pvals_all | 0.25 | 0.7978 | 0.7837 | 0.8134 | 0.7823 | 0.2898 | 0.9003 |
| H34x | consensus_pvals_all | 0.3 | 0.7648 | 0.7657 | 0.7638 | 0.7658 | 0.251 | 0.8885 |
| H35x | core-substituent_fps | 0.1 | 0.8212 | 0.8259 | 0.8147 | 0.8277 | 0.5103 | 0.8779 |
| H35x | core-substituent_fps | 0.15 | 0.79 | 0.7832 | 0.7994 | 0.7805 | 0.4407 | 0.8645 |
| H35x | core-substituent_fps | 0.2 | 0.7469 | 0.7528 | 0.7386 | 0.7553 | 0.376 | 0.8509 |
| H35x | core-substituent_fps | 0.25 | 0.7283 | 0.7268 | 0.7303 | 0.7262 | 0.343 | 0.8393 |
| H35x | core-substituent_fps | 0.3 | 0.7162 | 0.7127 | 0.7212 | 0.7112 | 0.325 | 0.8304 |
| H35x | deepsmiles_4_hashed_1024 | 0.1 | 0.8112 | 0.825 | 0.7909 | 0.8315 | 0.5146 | 0.8735 |
| H35x | deepsmiles_4_hashed_1024 | 0.15 | 0.7677 | 0.7788 | 0.7514 | 0.784 | 0.4273 | 0.8586 |
| H35x | deepsmiles_4_hashed_1024 | 0.2 | 0.7471 | 0.7524 | 0.7395 | 0.7548 | 0.3832 | 0.8465 |
| H35x | deepsmiles_4_hashed_1024 | 0.25 | 0.7226 | 0.7292 | 0.7131 | 0.7322 | 0.3434 | 0.8347 |
| H35x | deepsmiles_4_hashed_1024 | 0.3 | 0.7148 | 0.7214 | 0.7053 | 0.7243 | 0.3293 | 0.8257 |
| H35x | morgan2_hashed_1024 | 0.1 | 0.835 | 0.8264 | 0.848 | 0.822 | 0.5522 | 0.8810 |
| H35x | morgan2_hashed_1024 | 0.15 | 0.8007 | 0.7923 | 0.8131 | 0.7882 | 0.4788 | 0.8704 |
| H35x | morgan2_hashed_1024 | 0.2 | 0.766 | 0.764 | 0.7691 | 0.763 | 0.4189 | 0.8602 |
| H35x | morgan2_hashed_1024 | 0.25 | 0.7399 | 0.7351 | 0.747 | 0.7329 | 0.3678 | 0.8515 |
| H35x | morgan2_hashed_1024 | 0.3 | 0.7545 | 0.7535 | 0.7558 | 0.7531 | 0.3946 | 0.8478 |
| H35x | morgan4_hashed_1024 | 0.1 | 0.8234 | 0.8107 | 0.843 | 0.8039 | 0.5313 | 0.8811 |
| H35x | morgan4_hashed_1024 | 0.15 | 0.786 | 0.7727 | 0.8061 | 0.7659 | 0.4546 | 0.8668 |
| H35x | morgan4_hashed_1024 | 0.2 | 0.7554 | 0.7475 | 0.7671 | 0.7438 | 0.3971 | 0.8532 |
| H35x | morgan4_hashed_1024 | 0.25 | 0.7257 | 0.7251 | 0.7267 | 0.7247 | 0.3485 | 0.8410 |
| H35x | morgan4_hashed_1024 | 0.3 | 0.7242 | 0.7278 | 0.719 | 0.7294 | 0.3445 | 0.8333 |
| H35x | ngram_4_hashed_1024 | 0.1 | 0.8315 | 0.8274 | 0.838 | 0.8251 | 0.5604 | 0.8790 |
| H35x | ngram_4_hashed_1024 | 0.15 | 0.7951 | 0.7944 | 0.7962 | 0.7941 | 0.4774 | 0.8672 |
| H35x | ngram_4_hashed_1024 | 0.2 | 0.7738 | 0.7761 | 0.7706 | 0.7771 | 0.4286 | 0.8574 |
| H35x | ngram_4_hashed_1024 | 0.25 | 0.7575 | 0.7626 | 0.75 | 0.7649 | 0.402 | 0.8506 |
| H35x | ngram_4_hashed_1024 | 0.3 | 0.7749 | 0.7793 | 0.7686 | 0.7813 | 0.4342 | 0.8486 |
| H35x | ngram_4_hashed_256 | 0.1 | 0.8236 | 0.8178 | 0.8323 | 0.815 | 0.5258 | 0.8762 |
| H35x | ngram_4_hashed_256 | 0.15 | 0.787 | 0.788 | 0.7854 | 0.7886 | 0.4557 | 0.8625 |
| H35x | ngram_4_hashed_256 | 0.2 | 0.7686 | 0.7658 | 0.7726 | 0.7645 | 0.4183 | 0.8518 |
| H35x | ngram_4_hashed_256 | 0.25 | 0.748 | 0.7481 | 0.748 | 0.7481 | 0.3829 | 0.8429 |
| H35x | ngram_4_hashed_256 | 0.3 | 0.7595 | 0.7612 | 0.757 | 0.7619 | 0.4032 | 0.8398 |
| H35x | ngram_4_hashed_64 | 0.1 | 0.8243 | 0.8073 | 0.8511 | 0.7975 | 0.5369 | 0.8753 |
| H35x | ngram_4_hashed_64 | 0.15 | 0.7944 | 0.7801 | 0.8157 | 0.7731 | 0.4654 | 0.8644 |
| H35x | ngram_4_hashed_64 | 0.2 | 0.7671 | 0.7559 | 0.7837 | 0.7506 | 0.4167 | 0.8538 |
| H35x | ngram_4_hashed_64 | 0.25 | 0.7441 | 0.7318 | 0.762 | 0.7262 | 0.3718 | 0.8439 |
| H35x | ngram_4_hashed_64 | 0.3 | 0.7411 | 0.7299 | 0.7573 | 0.7249 | 0.3648 | 0.8377 |
| H35x | ngram_6_hashed_1024 | 0.1 | 0.821 | 0.8347 | 0.8 | 0.8419 | 0.5463 | 0.8577 |
| H35x | ngram_6_hashed_1024 | 0.15 | 0.7787 | 0.7961 | 0.7525 | 0.8049 | 0.4588 | 0.8476 |
| H35x | ngram_6_hashed_1024 | 0.2 | 0.7539 | 0.7657 | 0.7363 | 0.7716 | 0.4066 | 0.8381 |
| H35x | ngram_6_hashed_1024 | 0.25 | 0.7319 | 0.7403 | 0.7195 | 0.7442 | 0.3593 | 0.8292 |
| H35x | ngram_6_hashed_1024 | 0.3 | 0.7409 | 0.7489 | 0.7293 | 0.7525 | 0.375 | 0.8245 |
| H35x | ngram_6_hashed_256 | 0.1 | 0.8275 | 0.8182 | 0.8418 | 0.8132 | 0.5407 | 0.8758 |
| H35x | ngram_6_hashed_256 | 0.15 | 0.7907 | 0.7892 | 0.7931 | 0.7884 | 0.4664 | 0.8622 |
| H35x | ngram_6_hashed_256 | 0.2 | 0.7691 | 0.7665 | 0.7729 | 0.7653 | 0.4216 | 0.8510 |
| H35x | ngram_6_hashed_256 | 0.25 | 0.7395 | 0.7369 | 0.7433 | 0.7358 | 0.3673 | 0.8412 |
| H35x | ngram_6_hashed_256 | 0.3 | 0.7443 | 0.7414 | 0.7484 | 0.7401 | 0.3734 | 0.8359 |
| H35x | ngram_6_hashed_64 | 0.1 | 0.8058 | 0.8039 | 0.8087 | 0.8029 | 0.4983 | 0.8507 |
| H35x | ngram_6_hashed_64 | 0.15 | 0.7747 | 0.778 | 0.7697 | 0.7796 | 0.4357 | 0.8387 |
| H35x | ngram_6_hashed_64 | 0.2 | 0.7395 | 0.7557 | 0.716 | 0.763 | 0.3749 | 0.8267 |
| H35x | ngram_6_hashed_64 | 0.25 | 0.7173 | 0.7339 | 0.6931 | 0.7415 | 0.3365 | 0.8164 |
| H35x | ngram_6_hashed_64 | 0.3 | 0.7039 | 0.7176 | 0.6841 | 0.7238 | 0.3128 | 0.8084 |
| H35x | ngramsPE_4_hashed_1024 | 0.1 | 0.8075 | 0.8025 | 0.8148 | 0.8001 | 0.4957 | 0.8574 |
| H35x | ngramsPE_4_hashed_1024 | 0.15 | 0.7676 | 0.7637 | 0.7733 | 0.7619 | 0.419 | 0.8430 |
| H35x | ngramsPE_4_hashed_1024 | 0.2 | 0.7392 | 0.7395 | 0.7389 | 0.7396 | 0.3709 | 0.8303 |
| H35x | ngramsPE_4_hashed_1024 | 0.25 | 0.7261 | 0.7182 | 0.7374 | 0.7147 | 0.3427 | 0.8191 |
| H35x | ngramsPE_4_hashed_1024 | 0.3 | 0.7161 | 0.7074 | 0.7288 | 0.7035 | 0.3242 | 0.8106 |
| H35x | SMILES_extnd_connect_fps_2_1024 | 0.1 | 0.8036 | 0.8138 | 0.788 | 0.8191 | 0.5061 | 0.8613 |
| H35x | SMILES_extnd_connect_fps_2_1024 | 0.15 | 0.7788 | 0.7831 | 0.7725 | 0.7852 | 0.4445 | 0.8481 |
| H35x | SMILES_extnd_connect_fps_2_1024 | 0.2 | 0.7475 | 0.7542 | 0.7377 | 0.7573 | 0.3894 | 0.8369 |
| H35x | SMILES_extnd_connect_fps_2_1024 | 0.25 | 0.728 | 0.7323 | 0.7216 | 0.7343 | 0.3499 | 0.8274 |
| H35x | SMILES_extnd_connect_fps_2_1024 | 0.3 | 0.7339 | 0.7328 | 0.7354 | 0.7323 | 0.3585 | 0.8224 |
| H35x | SMILES_extnd_connect_fps_4_1024 | 0.1 | 0.841 | 0.8444 | 0.8359 | 0.846 | 0.5668 | 0.8871 |
| H35x | SMILES_extnd_connect_fps_4_1024 | 0.15 | 0.8025 | 0.8043 | 0.8 | 0.805 | 0.4854 | 0.8775 |
| H35x | SMILES_extnd_connect_fps_4_1024 | 0.2 | 0.7758 | 0.7732 | 0.7795 | 0.772 | 0.4316 | 0.8678 |
| H35x | SMILES_extnd_connect_fps_4_1024 | 0.25 | 0.7537 | 0.7555 | 0.751 | 0.7563 | 0.3946 | 0.8591 |
| H35x | SMILES_extnd_connect_fps_4_1024 | 0.3 | 0.7573 | 0.7563 | 0.7588 | 0.7558 | 0.3991 | 0.8542 |
| H35x | selfies_4_hashed_1024 | 0.1 | 0.7916 | 0.792 | 0.7909 | 0.7923 | 0.4717 | 0.8647 |
| H35x | selfies_4_hashed_1024 | 0.15 | 0.7696 | 0.7596 | 0.7839 | 0.7552 | 0.4133 | 0.8503 |
| H35x | selfies_4_hashed_1024 | 0.2 | 0.739 | 0.7397 | 0.738 | 0.74 | 0.3643 | 0.8355 |
| H35x | selfies_4_hashed_1024 | 0.25 | 0.7158 | 0.7101 | 0.7241 | 0.7076 | 0.3235 | 0.8219 |
| H35x | selfies_4_hashed_1024 | 0.3 | 0.7015 | 0.6906 | 0.7172 | 0.6857 | 0.3005 | 0.8103 |
| H35x | consensus_cls_all | 0.1 | 0.8805 | 0.869 | 0.898 | 0.8629 | 0.6494 | NA |
| H35x | consensus_cls_all | 0.15 | 0.8232 | 0.8187 | 0.8301 | 0.8164 | 0.5317 | NA |
| H35x | consensus_cls_all | 0.2 | 0.7867 | 0.7754 | 0.8033 | 0.7702 | 0.4482 | NA |
| H35x | consensus_cls_all | 0.25 | 0.7749 | 0.7605 | 0.7957 | 0.7541 | 0.4232 | NA |
| H35x | consensus_cls_all | 0.3 | 0.7745 | 0.7597 | 0.7961 | 0.753 | 0.4224 | NA |
| H35x | consensus_pvals_all | 0.1 | 0.8995 | 0.89 | 0.9139 | 0.8852 | 0.6914 | 0.9429 |
| H35x | consensus_pvals_all | 0.15 | 0.8613 | 0.8498 | 0.8783 | 0.8442 | 0.5986 | 0.9300 |
| H35x | consensus_pvals_all | 0.2 | 0.8219 | 0.8173 | 0.8286 | 0.8151 | 0.5243 | 0.9165 |
| H35x | consensus_pvals_all | 0.25 | 0.7957 | 0.7866 | 0.8088 | 0.7826 | 0.4639 | 0.9045 |
| H35x | consensus_pvals_all | 0.3 | 0.7849 | 0.7791 | 0.7935 | 0.7764 | 0.4477 | 0.8968 |
| H36x01 | consensus_cls_all | 0.1 | 0.8745 | 0.8633 | 0.8929 | 0.8561 | 0.6559 | NA |
| H36x01 | consensus_cls_all | 0.15 | 0.8245 | 0.8207 | 0.8304 | 0.8186 | 0.54 | NA |
| H36x01 | consensus_cls_all | 0.2 | 0.7814 | 0.7711 | 0.7966 | 0.7662 | 0.4418 | NA |
| H36x01 | consensus_cls_all | 0.25 | 0.7589 | 0.7528 | 0.7678 | 0.7501 | 0.3993 | NA |
| H36x01 | consensus_cls_all | 0.3 | 0.7569 | 0.7536 | 0.7616 | 0.7521 | 0.3967 | NA |
| H36x01 | consensus_pvals_all | 0.1 | 0.8918 | 0.8811 | 0.9099 | 0.8737 | 0.6977 | 0.9321 |
| H36x01 | consensus_pvals_all | 0.15 | 0.8552 | 0.8505 | 0.8626 | 0.8479 | 0.606 | 0.9185 |
| H36x01 | consensus_pvals_all | 0.2 | 0.8157 | 0.8172 | 0.8134 | 0.818 | 0.5221 | 0.9052 |
| H36x01 | consensus_pvals_all | 0.25 | 0.785 | 0.7846 | 0.7857 | 0.7844 | 0.4521 | 0.8925 |
| H36x01 | consensus_pvals_all | 0.3 | 0.7647 | 0.7707 | 0.756 | 0.7735 | 0.4167 | 0.8826 |
| H36x01 | core-substituent_fps | 0.1 | 0.8073 | 0.7952 | 0.8261 | 0.7885 | 0.5038 | 0.8469 |
| H36x01 | core-substituent_fps | 0.15 | 0.775 | 0.7691 | 0.7836 | 0.7663 | 0.4316 | 0.8362 |
| H36x01 | core-substituent_fps | 0.2 | 0.747 | 0.7479 | 0.7457 | 0.7483 | 0.3821 | 0.8254 |
| H36x01 | core-substituent_fps | 0.25 | 0.7211 | 0.7285 | 0.7102 | 0.7319 | 0.3399 | 0.8150 |
| H36x01 | core-substituent_fps | 0.3 | 0.7094 | 0.7134 | 0.7036 | 0.7152 | 0.3179 | 0.8068 |
| H36x01 | deepsmiles_4_hashed_1024 | 0.1 | 0.8099 | 0.8053 | 0.8171 | 0.8027 | 0.517 | 0.8500 |
| H36x01 | deepsmiles_4_hashed_1024 | 0.15 | 0.7739 | 0.7724 | 0.7762 | 0.7717 | 0.4412 | 0.8395 |
| H36x01 | deepsmiles_4_hashed_1024 | 0.2 | 0.7476 | 0.7436 | 0.7534 | 0.7417 | 0.3859 | 0.8289 |
| H36x01 | deepsmiles_4_hashed_1024 | 0.25 | 0.7182 | 0.7155 | 0.7222 | 0.7143 | 0.3323 | 0.8185 |
| H36x01 | deepsmiles_4_hashed_1024 | 0.3 | 0.7121 | 0.7141 | 0.7092 | 0.715 | 0.3232 | 0.8110 |
| H36x01 | morgan2_hashed_1024 | 0.1 | 0.8307 | 0.8077 | 0.8673 | 0.7941 | 0.5473 | 0.8929 |
| H36x01 | morgan2_hashed_1024 | 0.15 | 0.7966 | 0.7836 | 0.8163 | 0.777 | 0.4754 | 0.8785 |
| H36x01 | morgan2_hashed_1024 | 0.2 | 0.7676 | 0.7646 | 0.772 | 0.7632 | 0.4222 | 0.8646 |
| H36x01 | morgan2_hashed_1024 | 0.25 | 0.7486 | 0.7432 | 0.7564 | 0.7407 | 0.3822 | 0.8540 |
| H36x01 | morgan2_hashed_1024 | 0.3 | 0.7482 | 0.7483 | 0.7479 | 0.7484 | 0.3836 | 0.8481 |
| H36x01 | morgan4_hashed_1024 | 0.1 | 0.8174 | 0.7941 | 0.8553 | 0.7795 | 0.5278 | 0.8816 |
| H36x01 | morgan4_hashed_1024 | 0.15 | 0.788 | 0.7682 | 0.818 | 0.7579 | 0.4585 | 0.8674 |
| H36x01 | morgan4_hashed_1024 | 0.2 | 0.7545 | 0.7457 | 0.7677 | 0.7414 | 0.3974 | 0.8541 |
| H36x01 | morgan4_hashed_1024 | 0.25 | 0.7349 | 0.7287 | 0.7439 | 0.7259 | 0.358 | 0.8422 |
| H36x01 | morgan4_hashed_1024 | 0.3 | 0.7224 | 0.7191 | 0.7271 | 0.7177 | 0.3378 | 0.8333 |
| H36x01 | ngram_4_hashed_1024 | 0.1 | 0.8302 | 0.8028 | 0.8765 | 0.7839 | 0.5571 | 0.8799 |
| H36x01 | ngram_4_hashed_1024 | 0.15 | 0.7918 | 0.7742 | 0.8196 | 0.7639 | 0.4768 | 0.8690 |
| H36x01 | ngram_4_hashed_1024 | 0.2 | 0.7643 | 0.7531 | 0.781 | 0.7475 | 0.4174 | 0.8583 |
| H36x01 | ngram_4_hashed_1024 | 0.25 | 0.7359 | 0.7315 | 0.7425 | 0.7293 | 0.3637 | 0.8474 |
| H36x01 | ngram_4_hashed_1024 | 0.3 | 0.7368 | 0.7391 | 0.7336 | 0.7401 | 0.3674 | 0.8409 |
| H36x01 | ngram_4_hashed_256 | 0.1 | 0.8054 | 0.788 | 0.8339 | 0.7768 | 0.5108 | 0.8652 |
| H36x01 | ngram_4_hashed_256 | 0.15 | 0.7767 | 0.7643 | 0.7962 | 0.7573 | 0.4478 | 0.8510 |
| H36x01 | ngram_4_hashed_256 | 0.2 | 0.7518 | 0.7393 | 0.7708 | 0.7328 | 0.3957 | 0.8398 |
| H36x01 | ngram_4_hashed_256 | 0.25 | 0.7332 | 0.7294 | 0.7389 | 0.7276 | 0.3588 | 0.8291 |
| H36x01 | ngram_4_hashed_256 | 0.3 | 0.7271 | 0.7251 | 0.7301 | 0.7241 | 0.3473 | 0.8220 |
| H36x01 | ngram_4_hashed_64 | 0.1 | 0.8083 | 0.8022 | 0.818 | 0.7986 | 0.5176 | 0.8561 |
| H36x01 | ngram_4_hashed_64 | 0.15 | 0.7649 | 0.7667 | 0.7621 | 0.7677 | 0.4281 | 0.8421 |
| H36x01 | ngram_4_hashed_64 | 0.2 | 0.7423 | 0.7464 | 0.7363 | 0.7483 | 0.3808 | 0.8295 |
| H36x01 | ngram_4_hashed_64 | 0.25 | 0.7199 | 0.7255 | 0.7118 | 0.728 | 0.3382 | 0.8182 |
| H36x01 | ngram_4_hashed_64 | 0.3 | 0.7024 | 0.7135 | 0.6864 | 0.7185 | 0.3091 | 0.8090 |
| H36x01 | ngram_6_hashed_1024 | 0.1 | 0.8194 | 0.8069 | 0.8399 | 0.7988 | 0.5404 | 0.8641 |
| H36x01 | ngram_6_hashed_1024 | 0.15 | 0.7871 | 0.775 | 0.8057 | 0.7685 | 0.4636 | 0.8555 |
| H36x01 | ngram_6_hashed_1024 | 0.2 | 0.7592 | 0.7477 | 0.7762 | 0.7422 | 0.404 | 0.8457 |
| H36x01 | ngram_6_hashed_1024 | 0.25 | 0.7358 | 0.7322 | 0.7411 | 0.7305 | 0.3632 | 0.8361 |
| H36x01 | ngram_6_hashed_1024 | 0.3 | 0.7234 | 0.7255 | 0.7203 | 0.7265 | 0.3424 | 0.8288 |
| H36x01 | ngram_6_hashed_256 | 0.1 | 0.8017 | 0.7931 | 0.8157 | 0.7877 | 0.5075 | 0.8547 |
| H36x01 | ngram_6_hashed_256 | 0.15 | 0.7706 | 0.7579 | 0.7902 | 0.7511 | 0.4325 | 0.8431 |
| H36x01 | ngram_6_hashed_256 | 0.2 | 0.7449 | 0.7383 | 0.7547 | 0.7351 | 0.3824 | 0.8321 |
| H36x01 | ngram_6_hashed_256 | 0.25 | 0.7286 | 0.7266 | 0.7314 | 0.7257 | 0.35 | 0.8219 |
| H36x01 | ngram_6_hashed_256 | 0.3 | 0.7171 | 0.7198 | 0.7133 | 0.7209 | 0.3314 | 0.8142 |
| H36x01 | ngram_6_hashed_64 | 0.1 | 0.7954 | 0.7861 | 0.8105 | 0.7803 | 0.4946 | 0.8401 |
| H36x01 | ngram_6_hashed_64 | 0.15 | 0.7728 | 0.7607 | 0.7915 | 0.754 | 0.4386 | 0.8298 |
| H36x01 | ngram_6_hashed_64 | 0.2 | 0.7438 | 0.7391 | 0.751 | 0.7366 | 0.3836 | 0.8200 |
| H36x01 | ngram_6_hashed_64 | 0.25 | 0.7162 | 0.7155 | 0.7173 | 0.7152 | 0.3324 | 0.8102 |
| H36x01 | ngram_6_hashed_64 | 0.3 | 0.6994 | 0.6993 | 0.6995 | 0.6992 | 0.3015 | 0.8017 |
| H36x01 | ngramsPE_4_hashed_1024 | 0.1 | 0.815 | 0.8026 | 0.8349 | 0.7952 | 0.5252 | 0.8632 |
| H36x01 | ngramsPE_4_hashed_1024 | 0.15 | 0.7795 | 0.767 | 0.7984 | 0.7606 | 0.444 | 0.8520 |
| H36x01 | ngramsPE_4_hashed_1024 | 0.2 | 0.7506 | 0.7412 | 0.7647 | 0.7365 | 0.39 | 0.8409 |
| H36x01 | ngramsPE_4_hashed_1024 | 0.25 | 0.7303 | 0.7222 | 0.7421 | 0.7184 | 0.3517 | 0.8301 |
| H36x01 | ngramsPE_4_hashed_1024 | 0.3 | 0.7175 | 0.7137 | 0.723 | 0.712 | 0.33 | 0.8214 |
| H36x01 | SMILES_extnd_connect_fps_2_1024 | 0.1 | 0.8143 | 0.8028 | 0.8327 | 0.7959 | 0.5259 | 0.8740 |
| H36x01 | SMILES_extnd_connect_fps_2_1024 | 0.15 | 0.7935 | 0.7801 | 0.8137 | 0.7733 | 0.4679 | 0.8615 |
| H36x01 | SMILES_extnd_connect_fps_2_1024 | 0.2 | 0.7655 | 0.7572 | 0.7776 | 0.7534 | 0.4118 | 0.8503 |
| H36x01 | SMILES_extnd_connect_fps_2_1024 | 0.25 | 0.7374 | 0.7344 | 0.7416 | 0.7331 | 0.3629 | 0.8400 |
| H36x01 | SMILES_extnd_connect_fps_2_1024 | 0.3 | 0.7269 | 0.724 | 0.7311 | 0.7227 | 0.346 | 0.8327 |
| H36x01 | SMILES_extnd_connect_fps_4_1024 | 0.1 | 0.8265 | 0.8211 | 0.8346 | 0.8184 | 0.5378 | 0.8798 |
| H36x01 | SMILES_extnd_connect_fps_4_1024 | 0.15 | 0.7852 | 0.7838 | 0.7872 | 0.7832 | 0.4548 | 0.8652 |
| H36x01 | SMILES_extnd_connect_fps_4_1024 | 0.2 | 0.7575 | 0.7594 | 0.7546 | 0.7603 | 0.4015 | 0.8535 |
| H36x01 | SMILES_extnd_connect_fps_4_1024 | 0.25 | 0.7373 | 0.7356 | 0.7399 | 0.7348 | 0.3637 | 0.8435 |
| H36x01 | SMILES_extnd_connect_fps_4_1024 | 0.3 | 0.7307 | 0.7309 | 0.7304 | 0.731 | 0.3533 | 0.8367 |
| H36x01 | selfies_4_hashed_1024 | 0.1 | 0.8047 | 0.7858 | 0.8347 | 0.7747 | 0.4995 | 0.8523 |
| H36x01 | selfies_4_hashed_1024 | 0.15 | 0.7678 | 0.7641 | 0.7734 | 0.7622 | 0.4252 | 0.8403 |
| H36x01 | selfies_4_hashed_1024 | 0.2 | 0.7346 | 0.7345 | 0.7348 | 0.7345 | 0.3631 | 0.8288 |
| H36x01 | selfies_4_hashed_1024 | 0.25 | 0.7142 | 0.7125 | 0.7166 | 0.7117 | 0.3264 | 0.8176 |
| H36x01 | selfies_4_hashed_1024 | 0.3 | 0.6988 | 0.6968 | 0.7017 | 0.6959 | 0.2984 | 0.8080 |
| H40x | consensus_cls_all | 0.1 | 0.8498 | 0.8491 | 0.8508 | 0.8487 | 0.6118 | NA |
| H40x | consensus_cls_all | 0.15 | 0.8167 | 0.8122 | 0.8241 | 0.8094 | 0.5372 | NA |
| H40x | consensus_cls_all | 0.2 | 0.7752 | 0.7688 | 0.7855 | 0.7649 | 0.4533 | NA |
| H40x | consensus_cls_all | 0.25 | 0.7489 | 0.7397 | 0.7637 | 0.7341 | 0.4048 | NA |
| H40x | consensus_cls_all | 0.3 | 0.748 | 0.7358 | 0.7674 | 0.7285 | 0.402 | NA |
| H40x | consensus_pvals_all | 0.1 | 0.8665 | 0.865 | 0.8689 | 0.864 | 0.6443 | 0.9050 |
| H40x | consensus_pvals_all | 0.15 | 0.8397 | 0.8368 | 0.8443 | 0.8351 | 0.584 | 0.8917 |
| H40x | consensus_pvals_all | 0.2 | 0.8104 | 0.8065 | 0.8165 | 0.8042 | 0.5205 | 0.8806 |
| H40x | consensus_pvals_all | 0.25 | 0.773 | 0.7696 | 0.7784 | 0.7676 | 0.4507 | 0.8700 |
| H40x | consensus_pvals_all | 0.3 | 0.7496 | 0.7479 | 0.7522 | 0.7469 | 0.4089 | 0.8603 |
| H40x | core-substituent_fps | 0.1 | 0.7756 | 0.7793 | 0.7697 | 0.7816 | 0.4643 | 0.8108 |
| H40x | core-substituent_fps | 0.15 | 0.7465 | 0.7527 | 0.7366 | 0.7565 | 0.4078 | 0.7997 |
| H40x | core-substituent_fps | 0.2 | 0.7197 | 0.7284 | 0.7057 | 0.7336 | 0.3592 | 0.7896 |
| H40x | core-substituent_fps | 0.25 | 0.6988 | 0.706 | 0.6874 | 0.7102 | 0.3218 | 0.7806 |
| H40x | core-substituent_fps | 0.3 | 0.6795 | 0.6866 | 0.6683 | 0.6908 | 0.2887 | 0.7724 |
| H40x | deepsmiles_4_hashed_1024 | 0.1 | 0.7964 | 0.7982 | 0.7935 | 0.7993 | 0.5034 | 0.8307 |
| H40x | deepsmiles_4_hashed_1024 | 0.15 | 0.7603 | 0.7647 | 0.7532 | 0.7674 | 0.4356 | 0.8196 |
| H40x | deepsmiles_4_hashed_1024 | 0.2 | 0.7362 | 0.7426 | 0.726 | 0.7464 | 0.3894 | 0.8091 |
| H40x | deepsmiles_4_hashed_1024 | 0.25 | 0.7141 | 0.7184 | 0.7072 | 0.7209 | 0.3486 | 0.7998 |
| H40x | deepsmiles_4_hashed_1024 | 0.3 | 0.6979 | 0.7004 | 0.694 | 0.7019 | 0.3197 | 0.7918 |
| H40x | morgan2_hashed_1024 | 0.1 | 0.818 | 0.8162 | 0.821 | 0.815 | 0.5476 | 0.8624 |
| H40x | morgan2_hashed_1024 | 0.15 | 0.7885 | 0.7846 | 0.7948 | 0.7822 | 0.4833 | 0.8509 |
| H40x | morgan2_hashed_1024 | 0.2 | 0.7629 | 0.76 | 0.7675 | 0.7583 | 0.4343 | 0.8404 |
| H40x | morgan2_hashed_1024 | 0.25 | 0.7374 | 0.7373 | 0.7374 | 0.7373 | 0.3889 | 0.8309 |
| H40x | morgan2_hashed_1024 | 0.3 | 0.7356 | 0.7384 | 0.7312 | 0.74 | 0.3866 | 0.8249 |
| H40x | morgan4_hashed_1024 | 0.1 | 0.811 | 0.8077 | 0.8163 | 0.8057 | 0.5246 | 0.8608 |
| H40x | morgan4_hashed_1024 | 0.15 | 0.773 | 0.7682 | 0.7805 | 0.7654 | 0.4493 | 0.8465 |
| H40x | morgan4_hashed_1024 | 0.2 | 0.7437 | 0.7452 | 0.7414 | 0.7461 | 0.3988 | 0.8331 |
| H40x | morgan4_hashed_1024 | 0.25 | 0.7231 | 0.7237 | 0.7222 | 0.724 | 0.3613 | 0.8216 |
| H40x | morgan4_hashed_1024 | 0.3 | 0.7046 | 0.705 | 0.7039 | 0.7052 | 0.3297 | 0.8117 |
| H40x | ngram_4_hashed_1024 | 0.1 | 0.8152 | 0.8178 | 0.8111 | 0.8193 | 0.539 | 0.8520 |
| H40x | ngram_4_hashed_1024 | 0.15 | 0.7905 | 0.7892 | 0.7925 | 0.7885 | 0.4842 | 0.8421 |
| H40x | ngram_4_hashed_1024 | 0.2 | 0.7655 | 0.7638 | 0.7683 | 0.7627 | 0.4357 | 0.8335 |
| H40x | ngram_4_hashed_1024 | 0.25 | 0.7371 | 0.737 | 0.7373 | 0.737 | 0.3866 | 0.8254 |
| H40x | ngram_4_hashed_1024 | 0.3 | 0.7328 | 0.7353 | 0.7288 | 0.7367 | 0.3803 | 0.8201 |
| H40x | ngram_4_hashed_256 | 0.1 | 0.8128 | 0.8125 | 0.8133 | 0.8123 | 0.5346 | 0.8445 |
| H40x | ngram_4_hashed_256 | 0.15 | 0.7824 | 0.7819 | 0.7832 | 0.7816 | 0.4703 | 0.8349 |
| H40x | ngram_4_hashed_256 | 0.2 | 0.7506 | 0.7502 | 0.7512 | 0.75 | 0.4117 | 0.8259 |
| H40x | ngram_4_hashed_256 | 0.25 | 0.7311 | 0.7306 | 0.732 | 0.7303 | 0.3753 | 0.8176 |
| H40x | ngram_4_hashed_256 | 0.3 | 0.7197 | 0.7197 | 0.7196 | 0.7197 | 0.3561 | 0.8109 |
| H40x | ngram_4_hashed_64 | 0.1 | 0.8049 | 0.7968 | 0.8182 | 0.7916 | 0.5148 | 0.8396 |
| H40x | ngram_4_hashed_64 | 0.15 | 0.7776 | 0.7726 | 0.7857 | 0.7696 | 0.4613 | 0.8294 |
| H40x | ngram_4_hashed_64 | 0.2 | 0.7501 | 0.7462 | 0.7564 | 0.7439 | 0.4093 | 0.8200 |
| H40x | ngram_4_hashed_64 | 0.25 | 0.7228 | 0.7198 | 0.7276 | 0.7181 | 0.3601 | 0.8111 |
| H40x | ngram_4_hashed_64 | 0.3 | 0.7056 | 0.7015 | 0.7122 | 0.699 | 0.3306 | 0.8035 |
| H40x | ngram_6_hashed_1024 | 0.1 | 0.804 | 0.8052 | 0.8019 | 0.806 | 0.5158 | 0.8409 |
| H40x | ngram_6_hashed_1024 | 0.15 | 0.7764 | 0.7796 | 0.7711 | 0.7817 | 0.465 | 0.8306 |
| H40x | ngram_6_hashed_1024 | 0.2 | 0.7547 | 0.7589 | 0.7479 | 0.7614 | 0.4218 | 0.8214 |
| H40x | ngram_6_hashed_1024 | 0.25 | 0.7273 | 0.7341 | 0.7165 | 0.7382 | 0.3726 | 0.8130 |
| H40x | ngram_6_hashed_1024 | 0.3 | 0.7174 | 0.7242 | 0.7065 | 0.7284 | 0.3553 | 0.8068 |
| H40x | ngram_6_hashed_256 | 0.1 | 0.8011 | 0.7985 | 0.8053 | 0.797 | 0.507 | 0.8344 |
| H40x | ngram_6_hashed_256 | 0.15 | 0.775 | 0.7714 | 0.7808 | 0.7692 | 0.4569 | 0.8243 |
| H40x | ngram_6_hashed_256 | 0.2 | 0.7439 | 0.7455 | 0.7414 | 0.7464 | 0.4007 | 0.8150 |
| H40x | ngram_6_hashed_256 | 0.25 | 0.7186 | 0.7208 | 0.7149 | 0.7222 | 0.3559 | 0.8064 |
| H40x | ngram_6_hashed_256 | 0.3 | 0.6987 | 0.7011 | 0.6948 | 0.7026 | 0.3208 | 0.7981 |
| H40x | ngram_6_hashed_64 | 0.1 | 0.7954 | 0.7915 | 0.802 | 0.7889 | 0.5018 | 0.8212 |
| H40x | ngram_6_hashed_64 | 0.15 | 0.7625 | 0.7593 | 0.7676 | 0.7573 | 0.4366 | 0.8129 |
| H40x | ngram_6_hashed_64 | 0.2 | 0.7338 | 0.7342 | 0.7331 | 0.7344 | 0.3832 | 0.8042 |
| H40x | ngram_6_hashed_64 | 0.25 | 0.7142 | 0.7136 | 0.7151 | 0.7132 | 0.3474 | 0.7959 |
| H40x | ngram_6_hashed_64 | 0.3 | 0.6983 | 0.6951 | 0.7035 | 0.6931 | 0.319 | 0.7881 |
| H40x | ngramsPE_4_hashed_1024 | 0.1 | 0.7941 | 0.796 | 0.7909 | 0.7972 | 0.4981 | 0.8411 |
| H40x | ngramsPE_4_hashed_1024 | 0.15 | 0.7724 | 0.7727 | 0.772 | 0.7728 | 0.452 | 0.8283 |
| H40x | ngramsPE_4_hashed_1024 | 0.2 | 0.7404 | 0.7426 | 0.7368 | 0.744 | 0.3931 | 0.8176 |
| H40x | ngramsPE_4_hashed_1024 | 0.25 | 0.7173 | 0.7175 | 0.717 | 0.7176 | 0.3524 | 0.8080 |
| H40x | ngramsPE_4_hashed_1024 | 0.3 | 0.6975 | 0.6968 | 0.6986 | 0.6964 | 0.3171 | 0.7993 |
| H40x | SMILES_extnd_connect_fps_2_1024 | 0.1 | 0.7926 | 0.7911 | 0.7949 | 0.7902 | 0.4944 | 0.8213 |
| H40x | SMILES_extnd_connect_fps_2_1024 | 0.15 | 0.7624 | 0.761 | 0.7647 | 0.7601 | 0.4321 | 0.8118 |
| H40x | SMILES_extnd_connect_fps_2_1024 | 0.2 | 0.7392 | 0.7387 | 0.7401 | 0.7384 | 0.3884 | 0.8032 |
| H40x | SMILES_extnd_connect_fps_2_1024 | 0.25 | 0.7156 | 0.7161 | 0.7148 | 0.7164 | 0.3495 | 0.7949 |
| H40x | SMILES_extnd_connect_fps_2_1024 | 0.3 | 0.6947 | 0.6965 | 0.6918 | 0.6976 | 0.3136 | 0.7873 |
| H40x | SMILES_extnd_connect_fps_4_1024 | 0.1 | 0.8067 | 0.8006 | 0.8166 | 0.7968 | 0.5162 | 0.8439 |
| H40x | SMILES_extnd_connect_fps_4_1024 | 0.15 | 0.7697 | 0.7682 | 0.7722 | 0.7673 | 0.4481 | 0.8324 |
| H40x | SMILES_extnd_connect_fps_4_1024 | 0.2 | 0.7413 | 0.7402 | 0.7432 | 0.7395 | 0.394 | 0.8221 |
| H40x | SMILES_extnd_connect_fps_4_1024 | 0.25 | 0.7119 | 0.7153 | 0.7066 | 0.7172 | 0.3441 | 0.8118 |
| H40x | SMILES_extnd_connect_fps_4_1024 | 0.3 | 0.6989 | 0.7003 | 0.6965 | 0.7012 | 0.3203 | 0.8033 |
| H40x | selfies_4_hashed_1024 | 0.1 | 0.7848 | 0.7797 | 0.7929 | 0.7767 | 0.4724 | 0.8379 |
| H40x | selfies_4_hashed_1024 | 0.15 | 0.7585 | 0.7551 | 0.7637 | 0.7532 | 0.4193 | 0.8247 |
| H40x | selfies_4_hashed_1024 | 0.2 | 0.7384 | 0.7372 | 0.7404 | 0.7364 | 0.3859 | 0.8126 |
| H40x | selfies_4_hashed_1024 | 0.25 | 0.7147 | 0.7123 | 0.7184 | 0.7109 | 0.3462 | 0.8020 |
| H40x | selfies_4_hashed_1024 | 0.3 | 0.6948 | 0.6926 | 0.6982 | 0.6913 | 0.3126 | 0.7924 |
| H41x | consensus_cls_all | 0.1 | 0.8514 | 0.8518 | 0.8456 | 0.8572 | 0.7024 | NA |
| H41x | consensus_cls_all | 0.15 | 0.8074 | 0.8075 | 0.8025 | 0.8123 | 0.6146 | NA |
| H41x | consensus_cls_all | 0.2 | 0.7697 | 0.7697 | 0.7704 | 0.7691 | 0.5394 | NA |
| H41x | consensus_cls_all | 0.25 | 0.7478 | 0.7476 | 0.7572 | 0.7384 | 0.4956 | NA |
| H41x | consensus_cls_all | 0.3 | 0.7446 | 0.7443 | 0.7588 | 0.7303 | 0.4891 | NA |
| H41x | consensus_pvals_all | 0.1 | 0.8596 | 0.86 | 0.8549 | 0.8643 | 0.7185 | 0.8941 |
| H41x | consensus_pvals_all | 0.15 | 0.8278 | 0.828 | 0.8227 | 0.8329 | 0.6554 | 0.8815 |
| H41x | consensus_pvals_all | 0.2 | 0.7952 | 0.7953 | 0.7907 | 0.7996 | 0.5902 | 0.8700 |
| H41x | consensus_pvals_all | 0.25 | 0.7659 | 0.7658 | 0.7674 | 0.7643 | 0.5316 | 0.8597 |
| H41x | consensus_pvals_all | 0.3 | 0.7494 | 0.7493 | 0.7522 | 0.7466 | 0.4987 | 0.8514 |
| H41x | core-substituent_fps | 0.1 | 0.778 | 0.7779 | 0.7812 | 0.7748 | 0.5559 | 0.8133 |
| H41x | core-substituent_fps | 0.15 | 0.7499 | 0.7498 | 0.7528 | 0.7469 | 0.4996 | 0.8014 |
| H41x | core-substituent_fps | 0.2 | 0.7242 | 0.7241 | 0.7328 | 0.7156 | 0.4483 | 0.7908 |
| H41x | core-substituent_fps | 0.25 | 0.7109 | 0.7107 | 0.7207 | 0.7011 | 0.4217 | 0.7813 |
| H41x | core-substituent_fps | 0.3 | 0.689 | 0.6888 | 0.7004 | 0.6777 | 0.378 | 0.7728 |
| H41x | deepsmiles_4_hashed_1024 | 0.1 | 0.8114 | 0.8114 | 0.8117 | 0.811 | 0.6222 | 0.8426 |
| H41x | deepsmiles_4_hashed_1024 | 0.15 | 0.7787 | 0.7787 | 0.777 | 0.7803 | 0.5572 | 0.8330 |
| H41x | deepsmiles_4_hashed_1024 | 0.2 | 0.7513 | 0.7512 | 0.7546 | 0.7479 | 0.5024 | 0.8236 |
| H41x | deepsmiles_4_hashed_1024 | 0.25 | 0.7287 | 0.7286 | 0.732 | 0.7253 | 0.4572 | 0.8145 |
| H41x | deepsmiles_4_hashed_1024 | 0.3 | 0.7143 | 0.7141 | 0.7214 | 0.7071 | 0.4285 | 0.8072 |
| H41x | morgan2_hashed_1024 | 0.1 | 0.8278 | 0.828 | 0.8227 | 0.8329 | 0.6555 | 0.8643 |
| H41x | morgan2_hashed_1024 | 0.15 | 0.7932 | 0.7932 | 0.7915 | 0.7948 | 0.5863 | 0.8543 |
| H41x | morgan2_hashed_1024 | 0.2 | 0.7655 | 0.7655 | 0.764 | 0.767 | 0.531 | 0.8445 |
| H41x | morgan2_hashed_1024 | 0.25 | 0.7401 | 0.7401 | 0.7427 | 0.7375 | 0.4801 | 0.8352 |
| H41x | morgan2_hashed_1024 | 0.3 | 0.7429 | 0.7429 | 0.7421 | 0.7437 | 0.4858 | 0.8300 |
| H41x | morgan4_hashed_1024 | 0.1 | 0.8138 | 0.8142 | 0.805 | 0.8227 | 0.6277 | 0.8497 |
| H41x | morgan4_hashed_1024 | 0.15 | 0.7856 | 0.7855 | 0.7878 | 0.7834 | 0.5709 | 0.8396 |
| H41x | morgan4_hashed_1024 | 0.2 | 0.7589 | 0.7589 | 0.7602 | 0.7576 | 0.5177 | 0.8306 |
| H41x | morgan4_hashed_1024 | 0.25 | 0.7355 | 0.7354 | 0.7362 | 0.7347 | 0.4708 | 0.8222 |
| H41x | morgan4_hashed_1024 | 0.3 | 0.7328 | 0.7327 | 0.7362 | 0.7293 | 0.4654 | 0.8169 |
| H41x | ngram_4_hashed_1024 | 0.1 | 0.812 | 0.8129 | 0.7984 | 0.8257 | 0.6242 | 0.8467 |
| H41x | ngram_4_hashed_1024 | 0.15 | 0.782 | 0.7823 | 0.7746 | 0.7895 | 0.5641 | 0.8366 |
| H41x | ngram_4_hashed_1024 | 0.2 | 0.7557 | 0.756 | 0.7491 | 0.7623 | 0.5114 | 0.8271 |
| H41x | ngram_4_hashed_1024 | 0.25 | 0.7343 | 0.7343 | 0.7333 | 0.7353 | 0.4685 | 0.8184 |
| H41x | ngram_4_hashed_1024 | 0.3 | 0.7269 | 0.7268 | 0.7296 | 0.7241 | 0.4537 | 0.8123 |
| H41x | ngram_4_hashed_256 | 0.1 | 0.8154 | 0.8162 | 0.8048 | 0.826 | 0.6304 | 0.8484 |
| H41x | ngram_4_hashed_256 | 0.15 | 0.7852 | 0.7857 | 0.7762 | 0.7942 | 0.5701 | 0.8385 |
| H41x | ngram_4_hashed_256 | 0.2 | 0.7586 | 0.7589 | 0.7517 | 0.7655 | 0.5171 | 0.8291 |
| H41x | ngram_4_hashed_256 | 0.25 | 0.7329 | 0.7331 | 0.7237 | 0.7421 | 0.4658 | 0.8204 |
| H41x | ngram_4_hashed_256 | 0.3 | 0.7242 | 0.7243 | 0.7197 | 0.7287 | 0.4484 | 0.8138 |
| H41x | ngram_4_hashed_64 | 0.1 | 0.812 | 0.8125 | 0.8038 | 0.8203 | 0.6238 | 0.8420 |
| H41x | ngram_4_hashed_64 | 0.15 | 0.7747 | 0.7752 | 0.7652 | 0.7842 | 0.5494 | 0.8320 |
| H41x | ngram_4_hashed_64 | 0.2 | 0.7487 | 0.7488 | 0.7443 | 0.753 | 0.4972 | 0.8223 |
| H41x | ngram_4_hashed_64 | 0.25 | 0.7281 | 0.7281 | 0.7273 | 0.7289 | 0.4561 | 0.8130 |
| H41x | ngram_4_hashed_64 | 0.3 | 0.7131 | 0.7132 | 0.7119 | 0.7143 | 0.4262 | 0.8054 |
| H41x | ngram_6_hashed_1024 | 0.1 | 0.8092 | 0.8095 | 0.8047 | 0.8136 | 0.6175 | 0.8434 |
| H41x | ngram_6_hashed_1024 | 0.15 | 0.781 | 0.7813 | 0.7755 | 0.7865 | 0.5618 | 0.8328 |
| H41x | ngram_6_hashed_1024 | 0.2 | 0.7535 | 0.7536 | 0.7514 | 0.7557 | 0.5068 | 0.8232 |
| H41x | ngram_6_hashed_1024 | 0.25 | 0.728 | 0.728 | 0.7286 | 0.7273 | 0.4558 | 0.8141 |
| H41x | ngram_6_hashed_1024 | 0.3 | 0.7206 | 0.7205 | 0.7246 | 0.7166 | 0.4411 | 0.8079 |
| H41x | ngram_6_hashed_256 | 0.1 | 0.8054 | 0.8056 | 0.8021 | 0.8086 | 0.61 | 0.8342 |
| H41x | ngram_6_hashed_256 | 0.15 | 0.7746 | 0.7748 | 0.7703 | 0.779 | 0.549 | 0.8241 |
| H41x | ngram_6_hashed_256 | 0.2 | 0.755 | 0.7549 | 0.7564 | 0.7535 | 0.5097 | 0.8154 |
| H41x | ngram_6_hashed_256 | 0.25 | 0.731 | 0.731 | 0.7316 | 0.7305 | 0.462 | 0.8072 |
| H41x | ngram_6_hashed_256 | 0.3 | 0.7183 | 0.7183 | 0.7213 | 0.7154 | 0.4366 | 0.8006 |
| H41x | ngram_6_hashed_64 | 0.1 | 0.8014 | 0.8016 | 0.7988 | 0.804 | 0.6019 | 0.8278 |
| H41x | ngram_6_hashed_64 | 0.15 | 0.7704 | 0.7702 | 0.7748 | 0.7659 | 0.5401 | 0.8189 |
| H41x | ngram_6_hashed_64 | 0.2 | 0.7461 | 0.7461 | 0.7462 | 0.7461 | 0.492 | 0.8103 |
| H41x | ngram_6_hashed_64 | 0.25 | 0.7173 | 0.7174 | 0.716 | 0.7186 | 0.4345 | 0.8017 |
| H41x | ngram_6_hashed_64 | 0.3 | 0.6994 | 0.6994 | 0.6956 | 0.7031 | 0.3987 | 0.7941 |
| H41x | ngramsPE_4_hashed_1024 | 0.1 | 0.807 | 0.8075 | 0.7976 | 0.8163 | 0.6138 | 0.8442 |
| H41x | ngramsPE_4_hashed_1024 | 0.15 | 0.7744 | 0.7746 | 0.7706 | 0.7783 | 0.5487 | 0.8333 |
| H41x | ngramsPE_4_hashed_1024 | 0.2 | 0.7502 | 0.7504 | 0.744 | 0.7565 | 0.5004 | 0.8229 |
| H41x | ngramsPE_4_hashed_1024 | 0.25 | 0.726 | 0.7263 | 0.7169 | 0.7351 | 0.452 | 0.8134 |
| H41x | ngramsPE_4_hashed_1024 | 0.3 | 0.7129 | 0.713 | 0.7057 | 0.7201 | 0.4258 | 0.8060 |
| H41x | SMILES_extnd_connect_fps_2_1024 | 0.1 | 0.8005 | 0.8003 | 0.8033 | 0.7977 | 0.6005 | 0.8296 |
| H41x | SMILES_extnd_connect_fps_2_1024 | 0.15 | 0.7672 | 0.7672 | 0.767 | 0.7674 | 0.5342 | 0.8194 |
| H41x | SMILES_extnd_connect_fps_2_1024 | 0.2 | 0.7406 | 0.7405 | 0.7419 | 0.7392 | 0.481 | 0.8092 |
| H41x | SMILES_extnd_connect_fps_2_1024 | 0.25 | 0.7183 | 0.7183 | 0.7198 | 0.7169 | 0.4366 | 0.8000 |
| H41x | SMILES_extnd_connect_fps_2_1024 | 0.3 | 0.7016 | 0.7015 | 0.7066 | 0.6967 | 0.4031 | 0.7922 |
| H41x | SMILES_extnd_connect_fps_4_1024 | 0.1 | 0.8074 | 0.8075 | 0.8047 | 0.8102 | 0.6147 | 0.8438 |
| H41x | SMILES_extnd_connect_fps_4_1024 | 0.15 | 0.7772 | 0.7772 | 0.7774 | 0.7769 | 0.5542 | 0.8322 |
| H41x | SMILES_extnd_connect_fps_4_1024 | 0.2 | 0.7512 | 0.7512 | 0.7531 | 0.7493 | 0.5023 | 0.8222 |
| H41x | SMILES_extnd_connect_fps_4_1024 | 0.25 | 0.7254 | 0.7254 | 0.7255 | 0.7253 | 0.4508 | 0.8127 |
| H41x | SMILES_extnd_connect_fps_4_1024 | 0.3 | 0.7109 | 0.7109 | 0.712 | 0.7097 | 0.4217 | 0.8051 |
| H41x | selfies_4_hashed_1024 | 0.1 | 0.7995 | 0.7996 | 0.7962 | 0.8028 | 0.5988 | 0.8348 |
| H41x | selfies_4_hashed_1024 | 0.15 | 0.7693 | 0.7694 | 0.7645 | 0.7742 | 0.5386 | 0.8239 |
| H41x | selfies_4_hashed_1024 | 0.2 | 0.7494 | 0.7495 | 0.7443 | 0.7545 | 0.4987 | 0.8141 |
| H41x | selfies_4_hashed_1024 | 0.25 | 0.7274 | 0.7275 | 0.7246 | 0.7302 | 0.4548 | 0.8054 |
| H41x | selfies_4_hashed_1024 | 0.3 | 0.7112 | 0.7113 | 0.7093 | 0.7132 | 0.4224 | 0.7981 |

a. ROC_auc not available (NA) since consensus assignments were performed on classes and not p-value differences (p_value_H-statement_class – p_value_non-H-statement_class).
